# Mechanism of Action of Flavin-Dependent Halogenases

**DOI:** 10.1021/acscatal.2c05231

**Published:** 2022-11-30

**Authors:** Rhys D. Barker, Yuqi Yu, Leonardo De Maria, Linus O. Johannissen, Nigel S. Scrutton

**Affiliations:** † Manchester Institute of Biotechnology, The University of Manchester, 131 Princess Street, Manchester M1 7DN, U.K.; ‡ Research and Early Development, Respiratory & Immunology, BioPharmaceuticals R&D, AstraZeneca, Gothenburg 43283, Sweden

**Keywords:** DFT, molecular dynamics, cluster models, enzyme mechanism, halogenation, chlorination

## Abstract

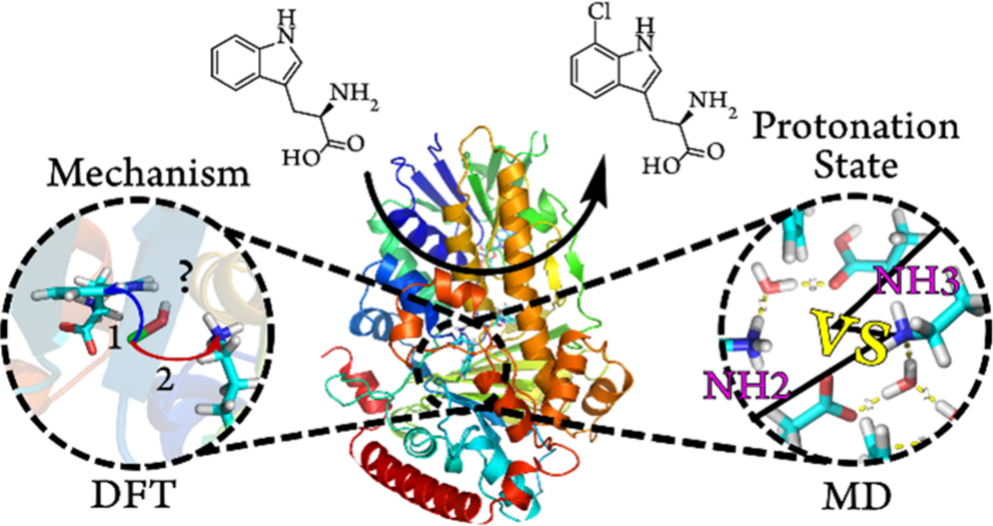

To rationally engineer the substrate scope and selectivity of flavin-dependent halogenases (FDHs), it is essential to first understand the reaction mechanism and substrate interactions in the active site. FDHs have long been known to achieve regioselectivity through an electrophilic aromatic substitution at C7 of the natural substrate Trp, but the precise role of a key active-site Lys residue remains ambiguous. Formation of hypochlorous acid (HOCl) at the cofactor-binding site is achieved by the direct reaction of molecular oxygen and a single chloride ion with reduced FAD and flavin hydroxide, respectively. HOCl is then guided 10 Å into the halogenation active site. Lys79, located in this site, has been proposed to direct HOCl toward Trp C7 through hydrogen bonding or a direct reaction with HOCl to form an −NH_2_Cl^+^ intermediate. Here, we present the most likely mechanism for halogenation based on molecular dynamics (MD) simulations and active-site density functional theory “cluster” models of FDH PrnA in complex with its native substrate l-tryptophan, hypochlorous acid, and the FAD cofactor. MD simulations with different protonation states for key active-site residues suggest that Lys79 directs HOCl through hydrogen bonding, which is confirmed by calculations of the reaction profiles for both proposed mechanisms.

## 1 Introduction

Enzymes can have diverse reaction scopes, but others are more limited, requiring intervention measures such as structure-based mutagenesis and directed evolution to expand their catalytic utility.^1^ There are indisputable benefits to using biocatalytic approaches in small molecule synthesis when contrasted with traditional synthetic approaches. These include increased atom efficiency, avoidance of waste streams, and use of less energy-intensive approaches to synthesis.^2−9^ For many industries, biocatalysis enables synthesis under milder reaction conditions (pH, temperature, and pressure), without functional group activation. Furthermore, the opportunity to replace subsequent synthetic steps with biocatalytic cascades—often in a “one pot” format—can enhance the operational and sustainability benefits of synthetic approaches.^10,11^ Consequently, biocatalytic cascades are increasingly being implemented in pharmaceutical and fine chemical manufacturing processes, for example, as a means of producing active pharmaceutical ingredients (APIs).^12−19^

Halogenation of drug compounds often leads to improved pharmacokinetic properties and bioactivity. This has resulted in a rapidly increasing number of halogenated APIs. Halogenated compounds make up approximately 25%^20−22^ of drugs sold on the market. Flavin-dependent halogenases (FDHs) are one of the three enzyme families that belong to the electrophilic halogenase class of enzymes. Heme-dependent, vanadate-dependent, and FDH enzymes all perform nucleophilic aromatic substitution, S_N_Ar, reactions, which utilize hypohalite (XO^–^) to produce a formally charged X^+^ species, which in turn reacts with substrate aromatic groups. FDHs have been shown to act with specificity and regioselectivity, making them potentially useful biocatalysts in the specific synthesis of halogenated products.^21^ There are to date at least 33 structurally characterized halogenases, of which 16 are members of the FDH family.

FDHs have been successfully engineered to have increased thermostability, substrate tolerance, and conversion of non-natural substrates.^23^ In particular, there has been notable success in expanding the scope of RebH^24^ to include sterically larger substrates. RebH was originally thought to be active with substrates that contain indole rings, similar to the natural substrate Trp.^24^ It has since been demonstrated that RebH has a wider substrate scope, but RebH,^24^ PrnA,^11^ and Thal^25^ still have a strong preference for indole-/pyrrole-containing substrates. FDHs facilitate halogenation at specific locations of the indole ring, with PrnA^11^ and RebH^24^ halogenating C7, whereas Thal^25^ and pyrH^26^ prefer C6 and C5, respectively. FDHs are therefore capable of regioselectively chlorinating at different substrate positions. This makes them promising biocatalysts for API biosynthesis.

Despite this promise, issues remain that hinder the widespread use of FDHs in API production, notably the activity and conversion rates of FDHs with pyrrole-like substrates that contain electron-withdrawing groups (EWGs). FDHs have lower conversion rates for electronically poor substrates, which is a major problem for target APIs that contain EWGs.^27^

**Figure 1:**
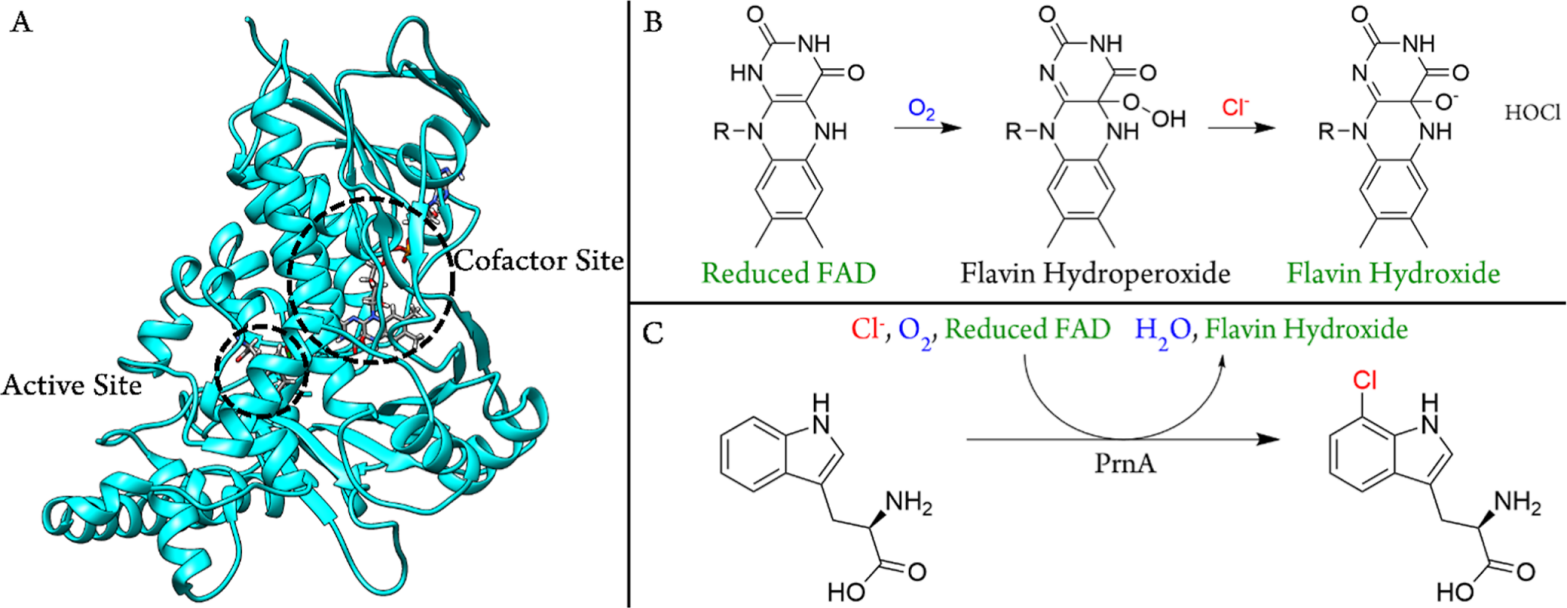
(A) Crystal structure of PrnA with cofactor flavin hydroxide and bound Trp and HOCl in the active site, (B) oxidation of FADH2 to flavin hydroxide FADHOH, producing a hydroxide ion, which reacts directly with a free chloride ion to produce HOCl, and (C) after producing HOCl, PrnA regioselectivity chlorinates natural substrate Trp.

FDHs require a flavin reductase to generate the reduced flavin, FADH_2_,^28^ which binds in a solvent-exposed groove separate from the halogenation active site^11,20^ (Figure 1A). Following reduction by the reductase, FADH_2_ is then oxidized to an intermediary flavin hydroperoxide (FADHOOH, the source of OH^+^), which in turn reacts with Cl^–^ to form flavin hydroxide (FADHOH) and hypochlorous acid (HOCl) (Figure 1B). HOCl is then transferred through a 10 Å long channel to the substrate,^11,24^ where it has been proposed that HOCl reacts with a proximal lysine residue to form an −NH_2_Cl^+^ species.^29,30^ Others, however, have suggested that the lysine residue serves to direct HOCl through a hydrogen bonding interaction with HOCl.^31,32^

**Figure 2:**
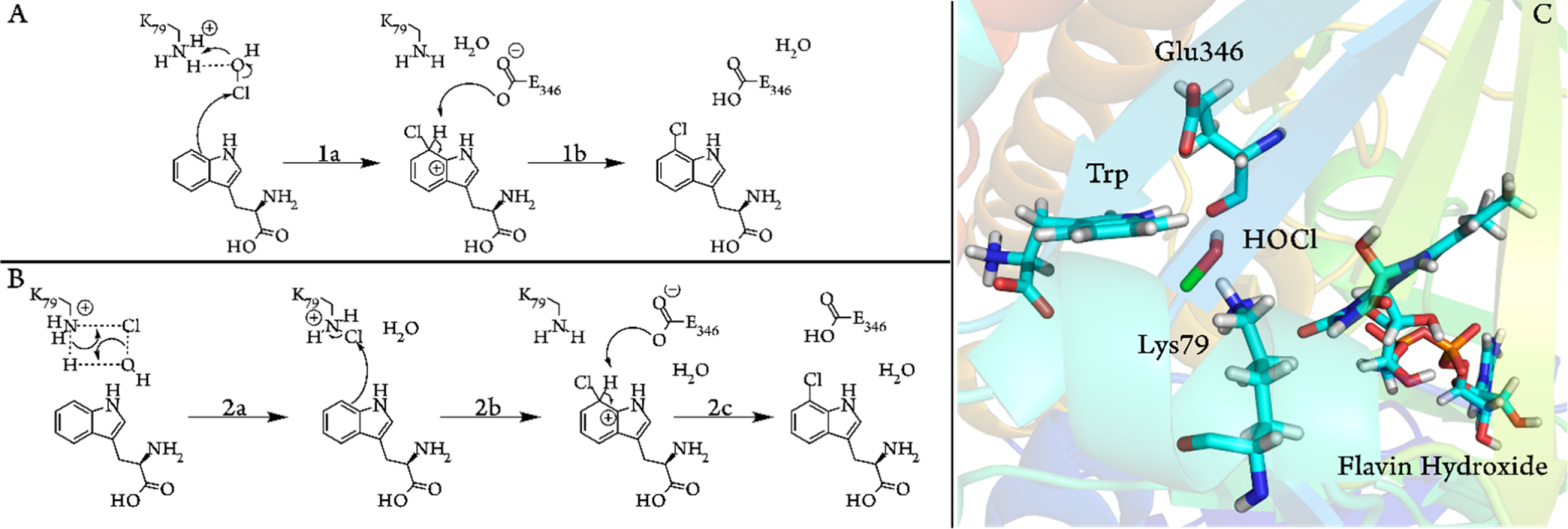
Proposed mechanisms for FDHs: (A) mechanism 1 is initiated by direct halogenation of substrate Trp by HOCl and (B) mechanism 2 is initiated by the formation of a lysinium intermediate. (C) PrnA crystal structure with modified reduced FAD (flavin hydroxide), HOCl, and substrate Trp. Positioning of HOCl with respect to Tryptophan, Glu346, and Lys79 was obtained from DFT geometry optimizations.

A mechanism of action for PrnA, a 7-tryptophan halogenase, was proposed following the determination of the X-ray crystal structure of the enzyme.^20^ This structure highlighted Lys79 as a crucial residue for activity, with a total loss of activity upon exchange with any other residue by site-directed mutagenesis.^33^ Glu346 was proposed to participate in the deprotonation of the so-called Wheland intermediate, leading to product formation (Figure 2 steps 1a and 2b).^11^ Subsequently, the discovery of a long-lived chlorolysine intermediate Lys-ϵNH-Cl in RebH, in another 7-tryptophan halogenase, led to the formulation of an alternative mechanism (Figure 2B).^29^ These mechanisms have both attracted great attention, and support for them has come from both experimental and theoretical studies. Here, these two mechanisms are referred to as mechanisms 1 and 2. PrnA and RebH have been the subjects of computational studies, each with contrasting roles proposed for the active site Lys. Calculations indicated that the chlorination step is rate-limiting in both mechanisms, with chlorination via the direct reaction of HOCl with Trp (mechanism 1) resulting in a barrier of 3.0 kcal mol^–1^,^32^ compared to chlorination via chloride transfer from chlorolysine −NH_2_Cl^+^ to Trp with a barrier of 3.5 kcal mol^–1^.^30^ However, the energy of formation of the critically important −NH_2_Cl^+^ intermediate has not been calculated, resulting in the publication of an incomplete energy profile for mechanism 2.^30^ Molecular dynamics (MD) simulations of −NH_2_Cl^+^ in RebH demonstrated that the −NH_2_Cl^+^ species orients toward C7 of Trp throughout the simulation time, consistent with the high regioselectivity observed with RebH.^29,34^

Herein, we use PrnA, a 7-tryptophan halogenase, to study the mechanism of action of halogenases in detail and to address the mechanistic uncertainty that remains in this family of enzymes. Prn was first characterized over 20 years ago.^35^ Since this time, it has been the subject of detailed substrate scope profiling, kinetic, spectroscopic, mutagenesis, and computational studies, making it a good target for further mechanistic study.^11,32,35−37^ We employ density functional theory (DFT) cluster models in combination with MD simulations to investigate both proposed mechanisms, beginning with the stability of bound HOCl and Trp in the enzyme active site and concluding with the calculation and comparison of energy profiles for mechanisms 1 and 2. Special consideration was paid to the protonation states of catalytic residues Lys79 and Glu346. The previous computational work^30,32^ on PrnA was performed with these two residues, proposed to be important in catalysis, modeled as a lysinium cation (ε-NH_3_^+^) and an unprotonated glutamate. Here, we consider alternative protonation states and provide a more comprehensive insight into the viability of proposed reaction mechanisms.

## 2 Methods

### 2.1 MD Simulations

Models were built based on the crystal structure of PrnA with the bound substrate 7-chlorotryptophan and reduced cofactor FAD (PDB code: 2AR8(6)). The substrate was modified to Trp, HOCl was added, and the reduced FAD cofactor was converted to flavin-hydroxide using UCSF Chimera.^38^ The protonation states of titratable residues were calculated using ProPKA3.^47^ HOCl was added using coordinates from energy-minimized DFT calculations. The Amber FF14SB force field was used, and general Amber force field^39^ parameters for HOCl and flavin-hydroxide were generated using Antechamber with charges obtained by RESP fitting^40^ to a HF/6-31G(d,p) single-point calculation on a structure optimized at the B3LYP/6-31G(d,p)//PCM(water) level of theory, using Gaussian09 revision D.^41^

The system was solvated in a TIP3P water box of at least 10 Å size around the protein, and counter-ions were added to neutralize the system charge. Calculations were then performed using Gromacs 2016.^42,43^ A stepwise energy minimization protocol was utilized, with a decreasing degree of positional restraints: (i) everything except the solvent and ions were restrained; (ii) restraints on hydrogen atoms were removed; and (iii) all restraints were removed.

After energy minimization, the solvent was equilibrated for 100 ps using the constant-volume *NVT* ensemble with positional restraints applied to the protein, cofactor, and substrates. The same progressive scheme of positional restraints as that during energy minimization was then applied to constant-pressure equilibration during successive 100 ps constant-pressure *NPT* ensemble simulations. Three 200 ns simulations were then performed on each complex, starting from the same equilibrated coordinates. In cases where equilibration leads to unstable HOCl binding (HOCl leaving the active site), hence biasing the production runs, equilibration was re-run, and the most stable *NPT* ensemble simulations chosen for production runs.

### 2.2 DFT Calculations

**Figure 3:**
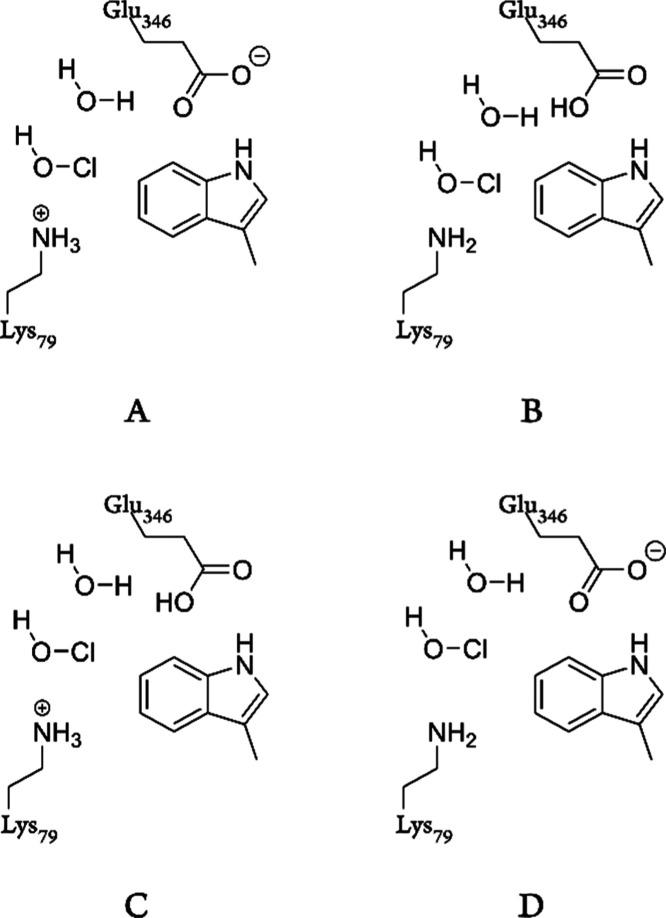
Cluster model 1 in four different protonation states.

Active site “cluster” models were constructed based on the same crystal structure as that of the MD simulations. Models of two different sizes were created: the smaller model (model 1) consists of truncated Trp, Glu346, Lys79, and a water molecule, which although not present in the crystal structure was added to facilitate the reaction (Figure 3 and S1). This model is not intended to reproduce the enzyme but is useful for comparing possible mechanisms. A larger, more representative model (model 2) includes the backbone of Glu346 and the surrounding aromatic and hydrogen bonding residues within 5 Å of Trp C7. Where the backbone lies beyond this range, side chains were truncated at the C-β, which was fixed during energy minimizations, otherwise the C-α was fixed (Figure S1). Due to the larger size of model 2, calculations using different protonation states for key mechanistic residues were performed on model 1, in order to investigate the specific effect of each residue and its protonation state on the mechanism. Thus, four versions of model 1 were created, representing each combination of potential protonation states for Lys79 and Glu346, with protonation state A identical to those of the aforementioned previous studies^30,32^ (Figure 3).

A much larger model (model 3; Figure S2) was created for barrier calculations of the most likely mechanism and protonation state, as determined from the MD simulations and smaller models, using a representative structure from the MD simulations. The structure with the smallest root mean square deviation (rmsd) for residues with at least one atom within 7 Å of the substrate Trp relative to the average structure across all simulations (for the relevant protonation state) was selected. For this model, all residues within the first sphere around Trp, HOCl, and the amino group of Lys79 were selected: Thr50, Ile51, Pro42, Ser53, Lys78, Ile81, His100, Leu101, Phe102, Gly103, Glu346, Ser347, Tyr443, Tyr444, Glu449, Phe454, Trp455, and Asn459. Three water molecules that formed a hydrogen bonding network between the backbone of substrate Trp, the backbone of Gly103, and the side chain of Asn459 were also included. Backbone atoms were included if involved in hydrogen bonding to other atoms in this selection, otherwise residues were truncated at the Cβ, and if a side chain was pointing out of the cluster model and not involved in any hydrogen bonding, the side chain was removed. One atom was kept fixed in each amino acid: the Cα if present, otherwise the Cβ. The final model consisted of 285 atoms, and the coordinates for the energy-minimized reactant state are available in Table S1.

Calculations were performed using Gaussian09^41^ for models 1 and 2 and Gaussian16^41^ for model 3, using the B3LYP^44^ functional. For models 1 and 2, the 6-31+G(d,p) basis sets were used for all atoms, and for model 3, 6-31+G(d,p) was used for the atoms directly involved in the reaction (HOCl, Trp C7–H, Glu 346 CO_2_^–^, and Lys79 NH_3_^+^), and 6-31G(d,p) was used for all other atoms. A polarizable continuum was applied to model a low dielectric environment (ε = 8.0), and Grimme’s D3 empirical dispersion correction was used.^45^ Relaxed scans were performed in order to model each chemical step, with a step size of no more than 0.10 Å. For model 1 and model 2, scans were performed along the forming bond. All barriers for model 1 were obtained using a singular scan along the bond forming axis, whereas for model 2, step 2a (formation of chloramine species—see Figure 2B), which involves the substitution of H for Cl on Lys79 and could in principle occur in either a stepwise or concerted manner, 2D scans were performed along both the forming N–Cl and O–H bonds. For model 3, scans were performed along a simple reaction coordinate defined as the difference between the breaking and forming bonds. The transition states were confirmed by the presence of a single imaginary frequency from frequency calculations, which were also used to compute the Gibbs free energies (including zero-point energy contributions and vibrational, but not configurational, entropy) for each chemical species.

## 3 Results and Discussion

### 3.1 MD Simulations

**Figure 4:**
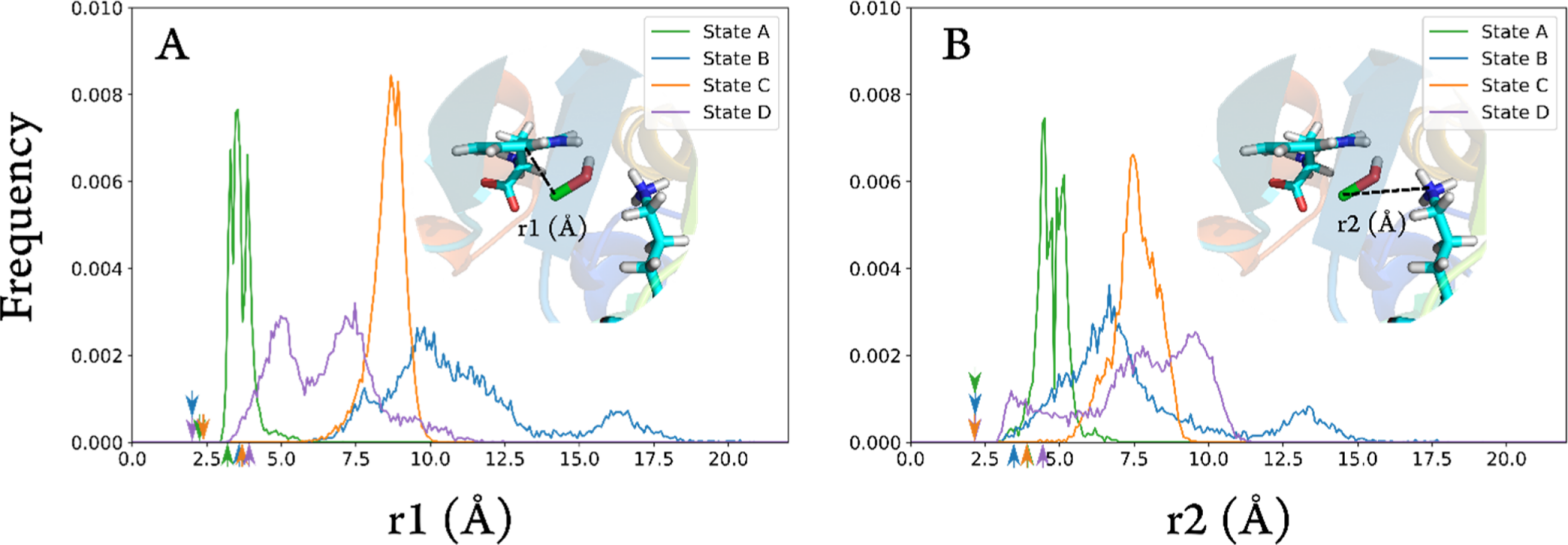
(A) Distributions of the average C7–Cl distance (r1) and (B) distribution of the average N–Cl distance (r2) of all MD runs of PrnA, state A (green), state B (orange), state C (blue), and state D (purple). The position on the histogram of each state’s respective DFT-calculated reactant r1 distance is indicated by arrows below the *x*-axis, with the TS r1 distances above the *x*-axis.

A series of MD simulations were carried out on PrnA with bound Trp, HOCl, and hydroxyl–FAD in order to explore the effect of Lys79 and Glu346 protonation states (Figure 3) on HOCl and Trp binding within the active site. The backbone rmsds relative to the energy-minimized starting structure and relative to the average structure for each protonation state are shown in Figures S3 and S4, respectively. For both mechanisms, it is essential that HOCl remains in the active site long enough for it to react, either directly with Trp for chlorination of C7 (mechanism 1) or with Lys79 to form the chlorolysine intermediate (mechanism 2). The distances of HOCl to Trp C7 and Lys79 N were therefore measured across all trajectories (Figures 4, S3, and S4), and it is clear that state A is the most favorable for either mechanism, with by far the shortest C7–Cl and N–Cl distances of any protonation state and equilibrium distances that overlap with the values in the DFT-optimized reactant states. Since the starting conformation was obtained from the DFT-optimized structures, with HOCl in the active site, one would expect a bias toward this geometry during the MD simulation. Therefore, it is clear that states B, C, and D are very ineffective at maintaining a reactive conformation compared to state A. For example, the distance distribution for state D has a small peak at a very short N–Cl distance (Figure 4B) because it takes ∼10 ns for the HOCl–Lys79 hydrogen bond to break during two of the three simulations (Figure S9), which suggests that, conversely, PrnA in protonation state D would also be very inefficient at “capturing” HOCl when it migrates into the active site after being produced at the flavin binding site.

**Figure 5:**
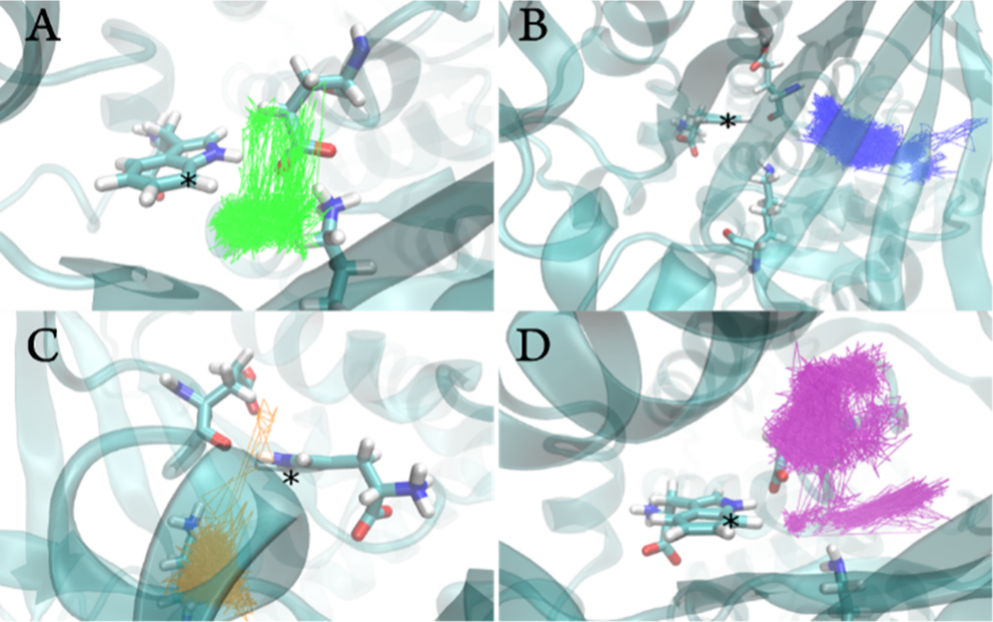
Pathways of the HOCl center of mass during MD simulations for protonation states A (green), B (blue), C (orange), and D (purple), shown on top of the representative positions of Trp, Glu346, and Lys79. Trp C7 is marked by an asterisk in all panels.

For states B, C, and D, HOCl is more loosely bound than for state A (Figure 5), with much larger rmsd values across the simulations relative to the starting structure (which was based on the position of HOCl in the DFT-optimized models). Trp is also more loosely bound for B and D but slightly more tightly bound in state C due to differences in hydrogen bonding (discussed below). The average HOCl/Trp rmsd values relative to their starting positions across all simulations are 1.17/1.32 Å (state A), 10.00/1.36 Å (state B), 1.58/0.87 Å (state C), and 5.63/1.27 Å (state D); see Figures S4 and S5. Note that in certain cases, the HOCl was found to leave the active site during the initial equilibration protocols, in which case the equilibration steps were rerun in order to begin the production runs with a bound HOCl. However, even biasing the production runs for HOCl binding in this manner, it is clear that state A is by far the protonation state that is the most compatible with catalysis by either proposed mechanism and that states C and D are particularly unfavorable; this is illustrated in Figure 5, which shows the pathways for the HOCl center of mass during the simulations.

Hydrogen bonding patterns change significantly between protonation states; the relative occurrence of specific hydrogen bonds is shown in Figures S10 and S11, Trp–PrnA interactions are illustrated in Figures S12 and S13, and HOCl–PrnA hydrogen bonds are illustrated in Figure S14. Specifically, the protonated Lys79 and deprotonated Glu346 are both essential for holding HOCl in place, and the deprotonated Glu346 is also key for Trp binding in the correct orientation for chlorination at C7. In state A, HOCl forms hydrogen bonds with Lys79, Ile82, Leu345, and Glu346, which promotes proximity of HOCl to Trp, resulting in a modal C7–Cl distance of 3.81 Å (Figure 4A). It is interesting that while HOCl binding is the most stable in state A, Trp binding appears to be the most stable in state C, which has a smaller Trp rmsd (Figure S4) and some more prevalent hydrogen bonding (Figure S11). However, the resulting Trp conformation is unfavorable for C7 chlorination: the strong hydrogen bond between protonated Glu346 (70% occurrence across all simulations) causes a slight rotation of Trp, which allows for better hydrogen bonding to Glu450 and Tyr444 (Figure S11) but results in a significantly longer C7–Cl distance; in fact, in this case, the closest carbon atom is C2 (average distances: 6.76 and 8.25 Å for C2–Cl and C7–Cl, respectively; Figure S15).

### 3.2 DFT Calculations

**Figure 6:**
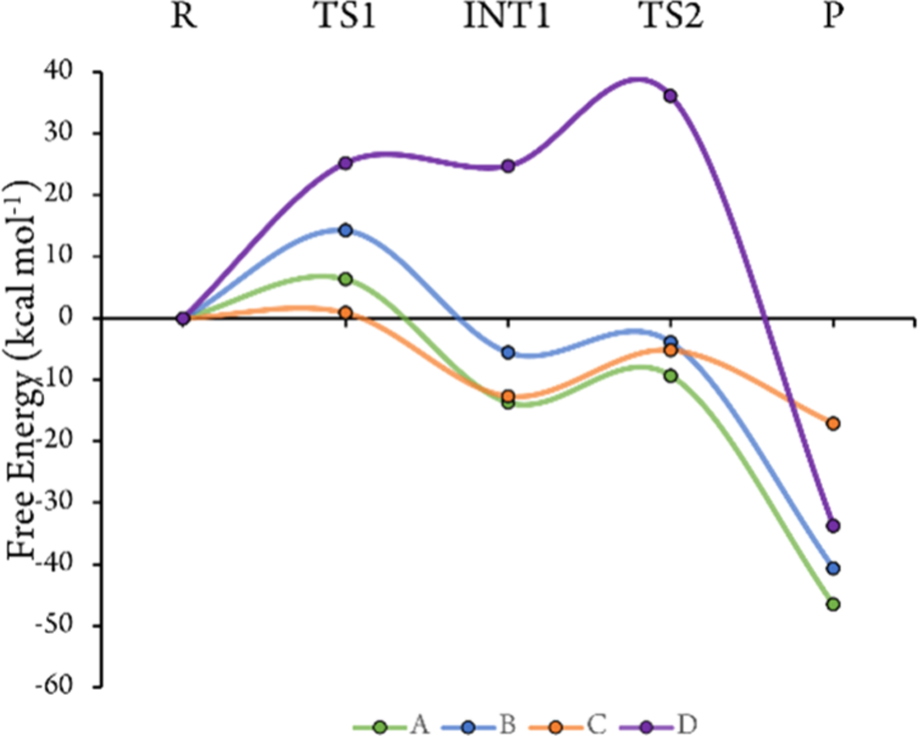
Energy profile for mechanism 1 with protonation states A (green), B (blue), C (red), and D (purple), calculated for model 1.

**Figure 7:**
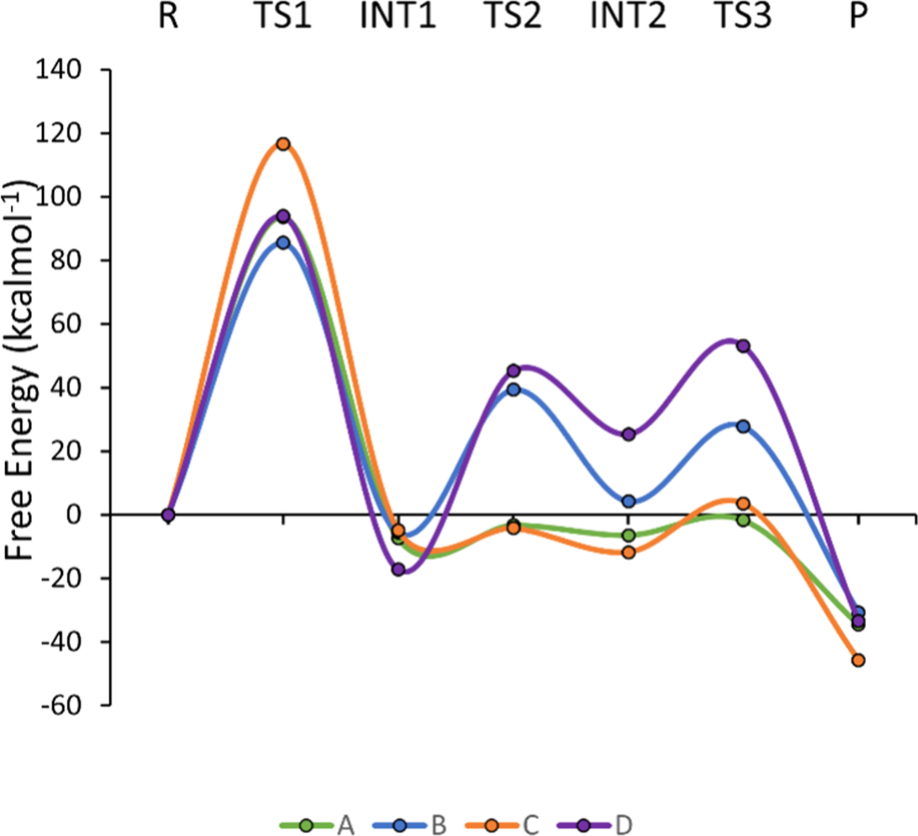
Energy profile for mechanism 2 with protonation states A (green), B (blue), C (red), and D (purple), calculated for model 1.

**Figure 8:**
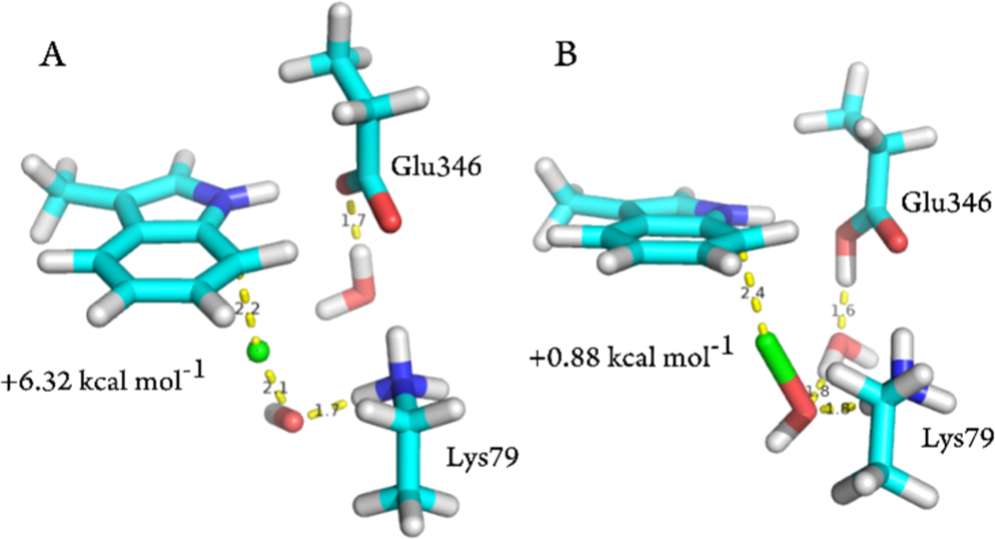
Comparison of hydrogen bonding in TS1 structures for mechanism 1 in model 1 between (A) state A and (B) state C.

DFT calculations using our smallest model, model 1, were performed to determine which mechanism is the most likely once HOCl has been “captured” by the PrnA active site. Due to the small size of this model, the results are only semi-quantitative but adequate for analyzing the fundamental differences between the two proposed mechanisms. This model cannot accurately capture properties such as the p*K*_a_ of Lys79, but by modifying the protonation state, this allows us to effectively study the effect of p*K*_a_ extremes, that is, a high-p*K*_a_ Lys is protonated, while a low-p*K*_a_ Lys is deprotonated. These calculations suggest that mechanism 1 (direct C7 chlorination) is much more likely than mechanism 2 (formation of the chlorolysine intermediate) since for every protonation state, the highest barrier for mechanism 1 is significantly lower than any barrier for mechanism 2 (Figures 6 and 7; all computed structures for both mechanisms are shown in Figures S12 and S13). To ensure that these results are not dependent on the choice of the dielectric constant, ε, single-point energy calculations were performed using a range of ε values (4, 8, and 20) as well as in a vacuum (Figures S18 and S19), and apart from the vacuum values, there is very little change. Note that for protonation states A and C (with protonated Lys79), we were unable to identify a proper transition state for this step: for the barriers shown in Figure 8 the highest-energy structures from potential energy scans for states A and C are very strained, with multiple imaginary frequencies of a similar magnitude, and attempts to obtain better transition state structures often resulted in C–N bond cleavage in Lys79 instead. This is not surprising as lysinium is inherently unreactive toward nucleophilic addition by HOCl (Figure S20), and chlorination of a protonated Lys79 would first require deprotonation to a neutral Lys79 in a separate chemical step (as opposed to a single chlorination–deprotonation step).

For mechanism 1, the largest barrier for all protonation states other than C is the chlorination of Trp C7, and the barrier height for this step depends on the strength of hydrogen bonding provided by Lys79: protonated Lys79 (states A and C) results in a lower barrier than deprotonated Lys79 (states B and D), since −NH_3_^+^ is a stronger hydrogen bond donor, which polarizes the O–Cl bond and increases the reactivity of HOC. The effect of hydrogen bonding on the O–Cl bond is illustrated in Figure S20. Lysinium has a much stronger effect than a neutral lysine, which has a very small effect on the reactivity of HOCl (in Figure S20, the effect of neutral lysine is negligible compared to that of lysinium). The active site water can also assist in hydrogen bonding to HOCl and has a stronger effect than neutral lysine, but even the combination of lysine plus a water molecule has a much smaller effect than lysinium. An additional water molecule can further enhance the effect of lysinium, which is why state C has the lowest barrier for TS1; the difference in hydrogen bonding in TS1 between states A and C is shown in Figure 8. This illustrates that from a protein engineering perspective, the key to improving the catalytic activity for mechanism 1 seems to lie in the abundance of hydrogen bonding to HOCl in the active site, which polarizes the O–Cl bond.

For protonation states A, B, and C, the Wheland intermediate formed by the initial addition of Cl^+^ at C7 (INT1 in Figure 6) is lower in energy than the reactant states. In state D, this is a high-energy intermediate due to the production of OH^–^ from HOCl, while in states A and C, the lysinium provides a proton to generate H_2_O, and in state B, the acid form of Glu346 provides this proton. Deprotonation of the Wheland intermediate during the next step (TS2) requires a deprotonated Glu346, which is why state D has a higher energy TS2 than the other three protonation states; for state B, TS2 is not elevated, despite also starting with a protonated Glu346, because Glu346 was deprotonated to form H_2_O with the OH^–^ from HOCl in the first step. Crucially, for all protonation states except D, the highest barrier is <15 kcal mol^–1^, and all produce a thermodynamically favorable final product. Therefore, while a protonated Lys79 facilitates chlorination of Trp C7, mechanism 1 also seems plausible with a neutral Lys79 if Glu346 is protonated. Note that changes in protonation states and hydrogen bonding during the reaction means that the Wheland intermediates obtained in mechanism 1 (INT1) are not necessarily identical to those obtained in mechanism 2 (INT2); for example, for state B, INT1 from mechanism 1 (Figure 6) is lower in energy than INT2 from mechanism 2 (Figure 7). However, this is a relatively small difference and does not affect the above discussion or the overall conclusions.

**Figure 9:**
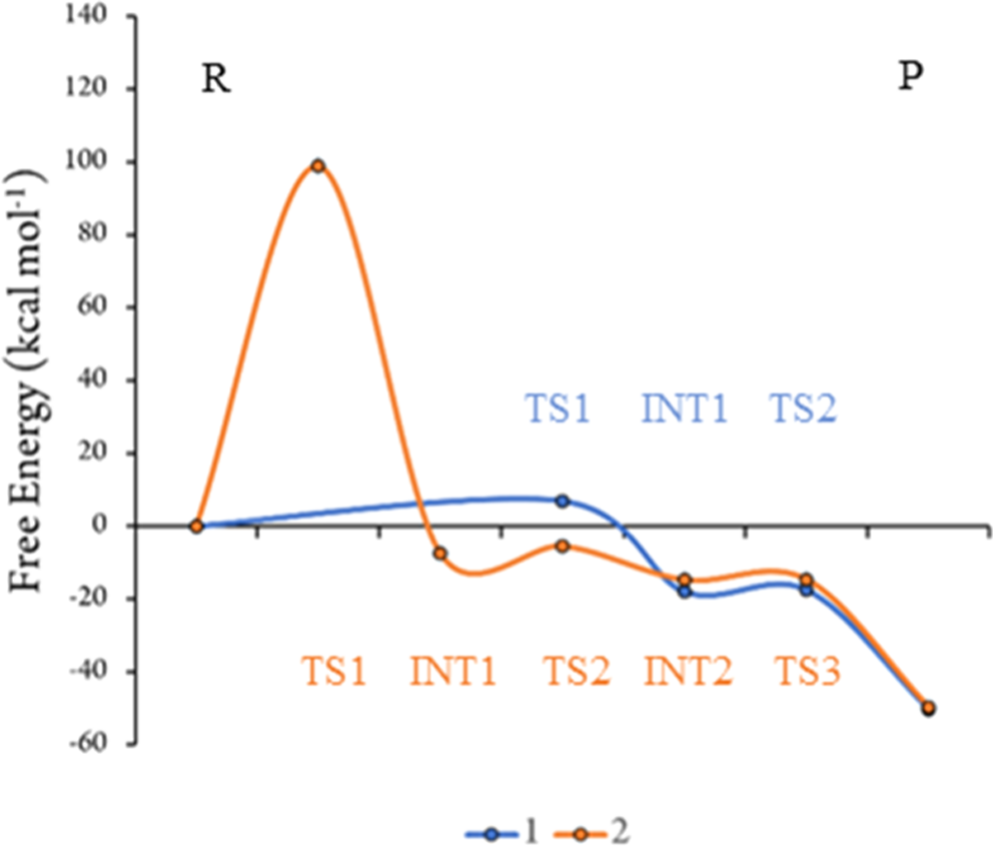
Free energy profile for mechanisms 1 and 2 for protonation state A, calculated for model 2.

Protonation state A was chosen for calculations with the larger model 2 since the MD simulations suggest that this is the best state for guiding HOCl into position for either of the two mechanisms. All structures for both mechanisms are shown in Figure S18. Crucially, the reaction profiles are very similar to those for model 1, with the first step as the largest in each mechanism (Figure 9), and formation of the chlorolysine leads to a much larger barrier for mechanism 2 (99 kcal mol^–1^) than that for mechanism 1 (9 kcal mol^–1^). Again, the specific choice of the dielectric constant does not affect the reaction profiles (Figure S22). The overall energy profile for mechanism 1 is very similar to that for model 1, despite the absence of the water molecule added to the smaller model to facilitate the reaction. This is because this water, which acts as a hydrogen bond acceptor to the water molecule formed from the protonation of OH^–^ by the Lys79 lysinium, is replaced by the backbone carbonyl of Glu346.

**Figure 10:**
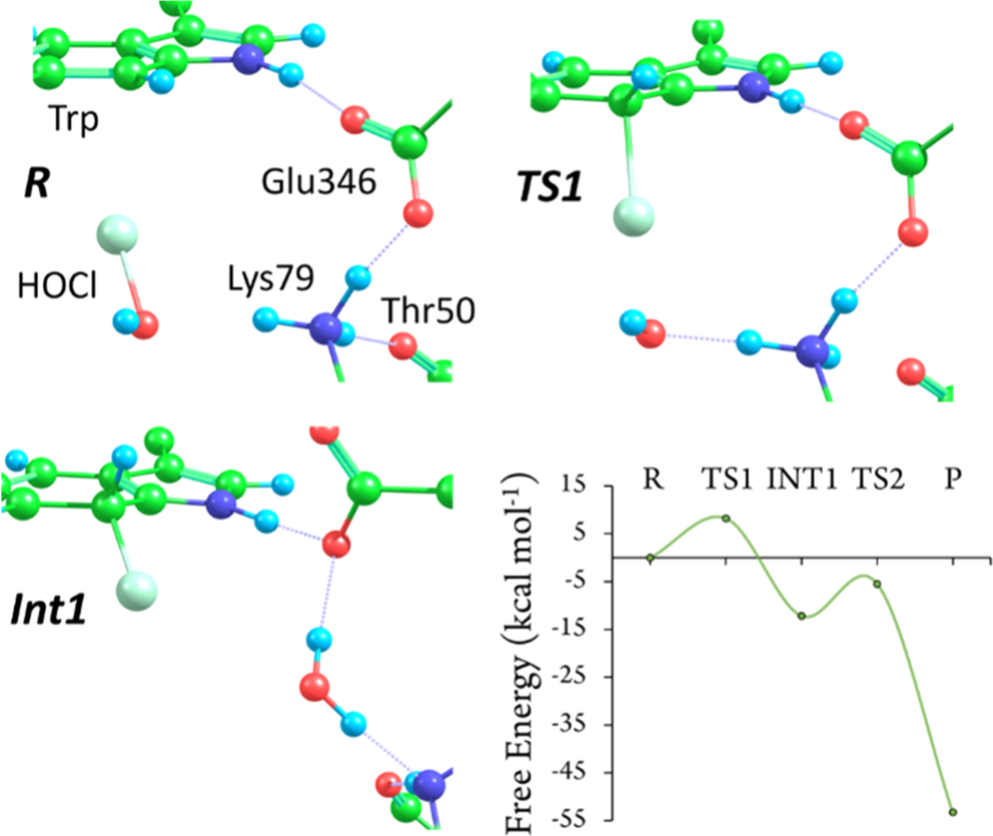
Close-up of structures from the chlorination of Trp C7 by HOCl in model 3, protonation state A of R, TS1, and Int1 for the first step and free energy profile for the entire reaction.

Finally, since mechanism 1 with protonation state A seems the most likely, we calculated the barriers for this mechanism using our largest model, model 3 (Figure 10). This produced a very similar reaction profile to that for model 2, with free energy barriers of 8 and 7 kcal mol^–1^ for the first and second steps, respectively. Since this model was created from a representative structure from the MD simulations of state A, structural changes that occur during the MD simulations do not lead to less reactive conformations. In reactant state R, Glu346 exhibits hydrogen bonding to both the substrate Trp −NH group and Lys79 −NH_3_^+^group. As Cl^+^ is transferred to Trp C7 and HOCl dissociates, Lys79 protonates the resulting OH^–^, which weakens the hydrogen bond between Lys79 and Glu346, allowing Glu346 to reposition itself and stabilize the Wheland intermediate, ready to deprotonate it. The water molecule formed from HOCl and a Lys79 proton now forms a hydrogen bond with the Glu346 carboxylate instead of its backbone carbonyl as in model 2. However, despite these differences, all the DFT calculations taken together indicate that chlorination of Trp most likely occurs from protonation state A via mechanism 1.

### 3.3 Broader Context

There is experimental evidence for the formation of a long-lived LysNHCl intermediate during enzymatic halogenation in RebH,^29^ which was shown to persist after the removal of FAD and is capable of chlorinating Trp with kinetically competent rates (although it would likely perform chlorination in the much more reactive LysNH_2_Cl^+^ form). However, LysNHCl was isolated in the absence of the Trp substrate, where the competing mechanism by direct chlorination of Trp (mechanism 1) cannot occur. (The enzyme was isolated on a desalting column, resulting in the nearly complete removal of FAD, and incubated at 25 °C for 30 min. L-[14C]Trp was then added in a second reaction.)^36^ ClTrp was produced in the absence of FAD, which did not occur in K79A and K79M variants. From this, they infer the formation of the chlorolysine species but were unable to isolate it via mass spectroscopy, crystallography, and so on. Further computational work revealed that LysNHCl specifically orients toward C7/C8 of the substrate in MalA′, which can explain the observed regioselectivity.^30^ The computed free energies of chlorination via mechanisms 1 and 2 as calculated by Karabencheva-Christova et al.^32^ and Fraley et al.,^30^ respectively, show very similar barriers for the chlorination of Trp, namely, 3.0 kcal mol^–1^ for direct chlorination by HOCl and 3.5 kcal mol^–1^ for chlorination by LysNH_2_Cl^+^. However, since the initial formation of LysNH_2_Cl^+^ (or LysNHCl) was not computed, a clear preference for either mechanism cannot be ascertained from these studies. Our calculations also suggest that HOCl and LysNH_2_Cl^+^ are similarly reactive toward Trp as once the chlorolysine intermediate is formed, the barriers for chlorination for each protonation state are comparable to those observed in mechanism 1, although our barriers are somewhat larger than those in the computational studies mentioned above. However, our calculations further suggest that the initial formation of the chlorolysine intermediate is much more unlikely than the direct chlorination of Trp C7. However, in the absence of Trp, formation of chlorolysine may operate as a protective mechanism.

A caveat of the smaller cluster models used in this study to rule out mechanism 2 is that while they allow us to analyze the fundamental chemistry of the two mechanisms of interest, they do not account for the specific electrostatic environment afforded by the broader protein environment. However, given the large differences in barrier heights between the two mechanisms, this would not affect the overall conclusions regarding the preference for direct chlorination of Trp by HOCl (mechanism 1). Additionally, increasing the reactivity of HOCl would facilitate both mechanisms, and while mechanism 2 could in principle be enhanced by a significantly more nucleophilic Lys79, our MD simulations suggest that Lys79 needs to be present in the non-nucleophilic lysinium state since this is required to bind HOCl, and there are no surrounding residues capable of deprotonating the lysinium prior to the reaction proper (Figure S24). It is also highly unlikely that a water molecule would deprotonate the lysinium: by definition, in protonation state A, Lys79 has a p*K*_a_ of > 7, and any stabilization of the positive charge that develops on Lys79 during the formation of the [Lys79-NH_2_Cl^δ+^...OH^δ−^] transition state, required to make this the preferred mechanism, would also stabilize the lysinium form of Lys79, which means that this would be an inefficient mechanism, especially given the proximity of HOCl to Trp C7 (Figure 4) and the relatively low barrier for direct chlorination of C7. Additionally, our MD simulations show very few water molecules in close proximity to Lys79 in protonation state A (Figure S25): there are no water molecules within 4, 6, or 8 Å of the Lys N in 70, 66, and 62% of the total simulation frames, respectively.

Our results agree with recent calculations on the halogenase Thal, which suggested that Lys79 has an unusually low p*K*_a_ of ∼7.5–7.6.^46^ The authors of the mentioned study suggested this might allow Lys79 to act as a proton donor during catalysis, which is indeed the case in our calculations. In fact, Lys79 performs two roles during catalysis via mechanism 1, and we propose that such a low p*K*_a_, just above 7, which allows it to remain protonated at neutral pH while acting as a stronger hydrogen bond donor than if it had a larger p*K*_a_, is ideal for both roles: (i) “catching” HOCl as it migrates in to the active site and (ii) enhancing the reactivity of HOCl by polarizing the O–Cl bond and later protonating the resulting OH^–^.

## 4 Conclusions

The mechanism for substrate halogenation in FDHs is contentious. There have been experimental and theoretical studies arguing for both the direct chlorination of the substrate by HOCl (mechanism 1) and the formation of a chlorolysine intermediate prior to the chlorination of the substrate (mechanism 2). We have studied both mechanisms using DFT calculations and MD simulations for chlorination of substrate Trp in the enzyme PrnA, where in principle the observed regioselectivity toward C7 chlorination could arise from hydrogen bonding guiding HOCl or from a long-lived −NH_2_Cl^+^ (or −NHCl) intermediate, formed by Lys79, orienting the chlorine toward C7. All four permutations of the Lys79 and Glu346 protonation states were used for MD simulations and small cluster model DFT calculations, which suggest that protonated Lys79 and deprotonated Glu346 are required for stable HOCl binding in the active site over the course of the simulations. As this is a prerequisite for both mechanisms, we estimate that it is the most likely protonation state. DFT calculations showed that this protonation state is also the preferential state for mechanism 1 as the strong hydrogen bond between Lys79 and HOCl enhances the reactivity of HOCl and allows it to protonate the OH^–^ formed by the dissociation of HOCl, while the carboxylate of Glu346 stabilizes and then deprotonates the Wheland intermediate formed. Mechanism 2 can be ruled out as the barriers are significantly higher than those for mechanism 1 for every protonation state. Taken together, these calculations provide evidence for the direct chlorination mechanism and the dual roles of key residues Lys79 and Glu346 in initial binding and during the chemical reaction.

## Supporting Information Available

Models 1 and 2 used in the DFT calculations in 2D and 3D forms; energy-minimized structure of the model 3 reactant; rmsd of the PrnA backbone during each MD simulation for protonation states A–D; rmsd of the PrnA backbone relative to the average structure for protonation states A–D; rmsd of the PrnA active site relative to the average structure for protonation states A–D; Trp C7–Cl distance versus time plots; Lys–Cl distance versus time plots; rmsd values of substrate Trp and HOCl during each MD simulation for protonation states A–D after structural alignment of the PrnA backbone; average % occurrence of hydrogen bonding between HOCl and PrnA and Trp and PrnA for all production runs for each protonation state; illustrations of hydrogen bonding; distributions of C2–Cl and C7–Cl distances during all MD runs of PrnA; structures from the reaction profile of mechanism 1 and mechanism 2; energy profiles of mechanism 1 and mechanism 2; effect of hydrogen bonding on O–Cl bond polarization and bond disassociation energy; reactivity of HOCl toward neutral and protonated lysine analogues and the tryptophan analogue; residues in the first sphere around Lys 79; number of water molecules within 4, 6, and 8 Å of Lys 79 N during three MD simulations of protonation state A; and Cartesian coordinates of the energy-minimized reactant state of model 3 (PDF)
