# Supplementary material for: Mechanism of Action of Flavin-Dependent Halogenases
Source: ACS Catal. 2022 Nov 30;12(24):15352–60. doi: 10.1021/acscatal.2c05231 (PMC9764358; doi:10.1021/acscatal.2c05231)
Supplement: Supplementary file 1 — cs2c05231_si_001.pdf [file cs2c05231_si_001.pdf]

Supporting Information

The Mechanism of Action in Flavin-Dependent Halogenases

Rhys D. Barker,[1] Yuqi Yu,[1] Leonardo De Maria,[2] Linus O. Johannissen,[1*] Nigel S. Scrutton.[1*]

[1]Manchester Institute of Biotechnology, The University of Manchester, 131 Princess Street, Manchester, M1 7DN, UK. [2]Research and Early Development, Respiratory & Immunology, BioPharmaceuticals R&D, AstraZeneca, Gothenburg, 43150 Sweden.

**Linus O. Johannissen – linus.johannissen@manchester.ac.uk*

**Nigel S. Scrutton – nigel.scrutton@manchester.ac.uk*

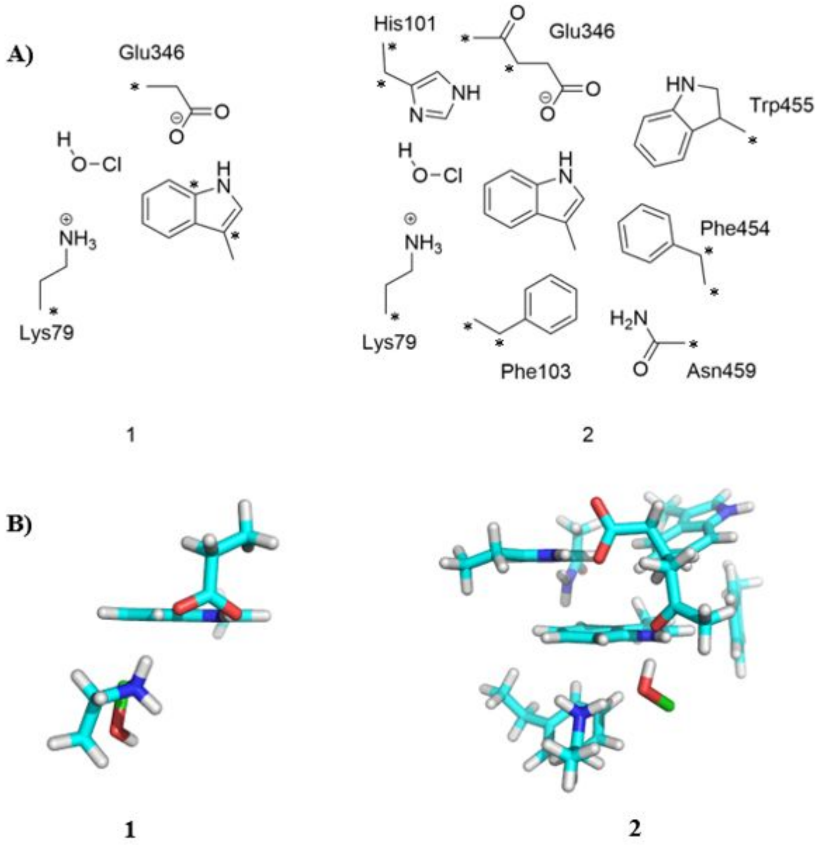

Figure S1. Models 1 and 2 used in DFT calculations represented in (A) 2D form and (B) visualised in 3D. * represents atoms kept fixed during energy minimizations.

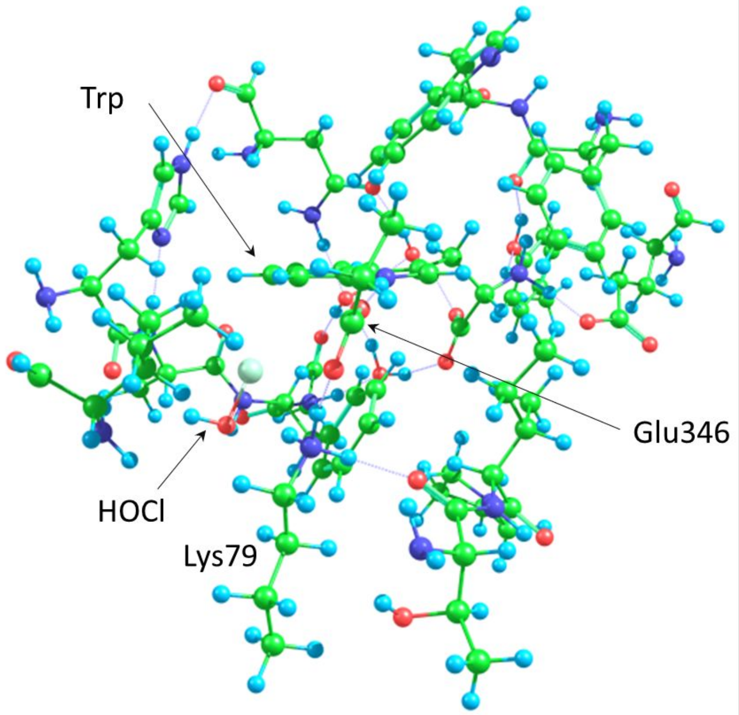

Figure S2. Energy minimised structure of Model 3 reactant.

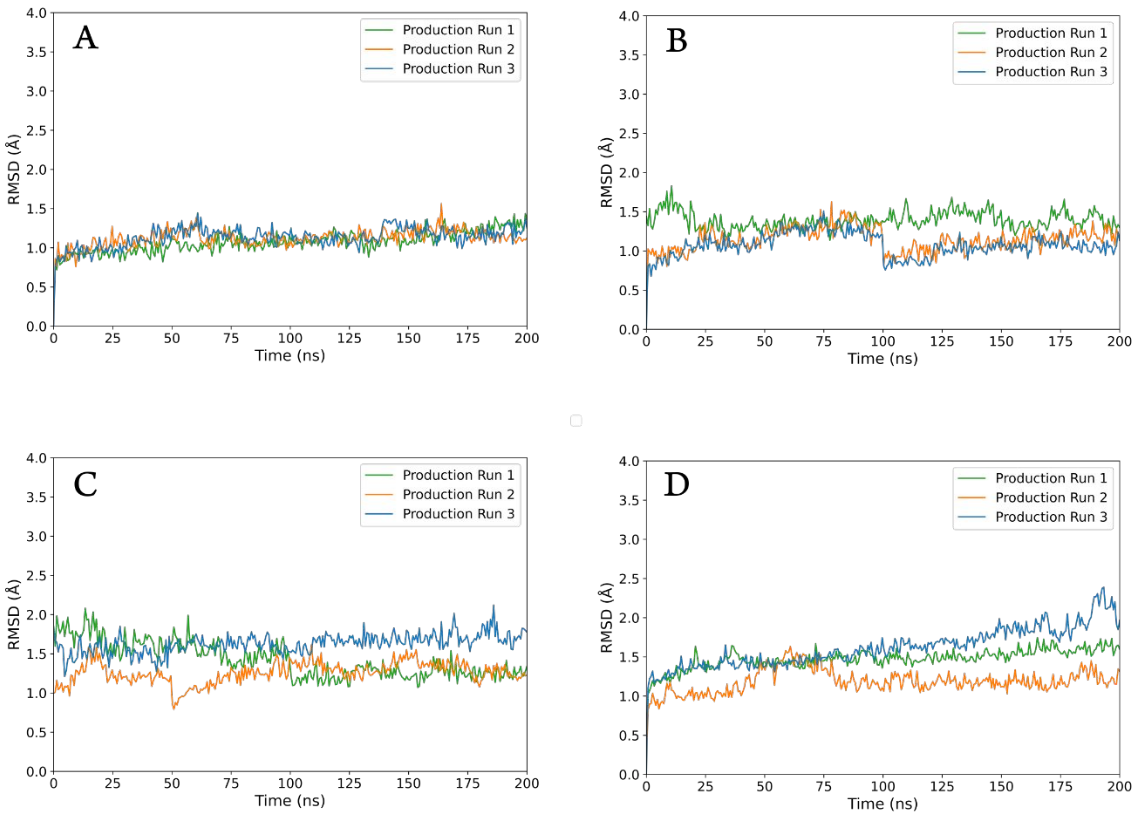

Figure S3. RMSD of PrnA backbone during each MD simulation for protonation states A-D.

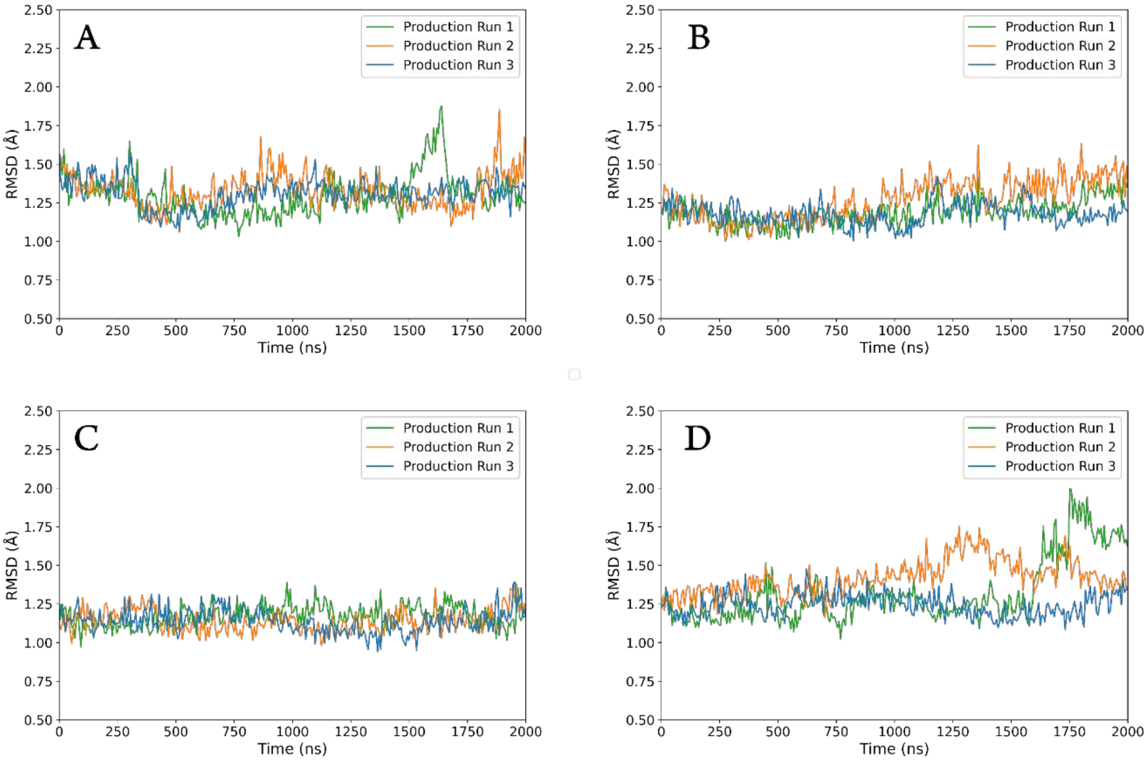

Figure S4. RMSD of PrnA backbone relative to the average structure for protonation states A-D.

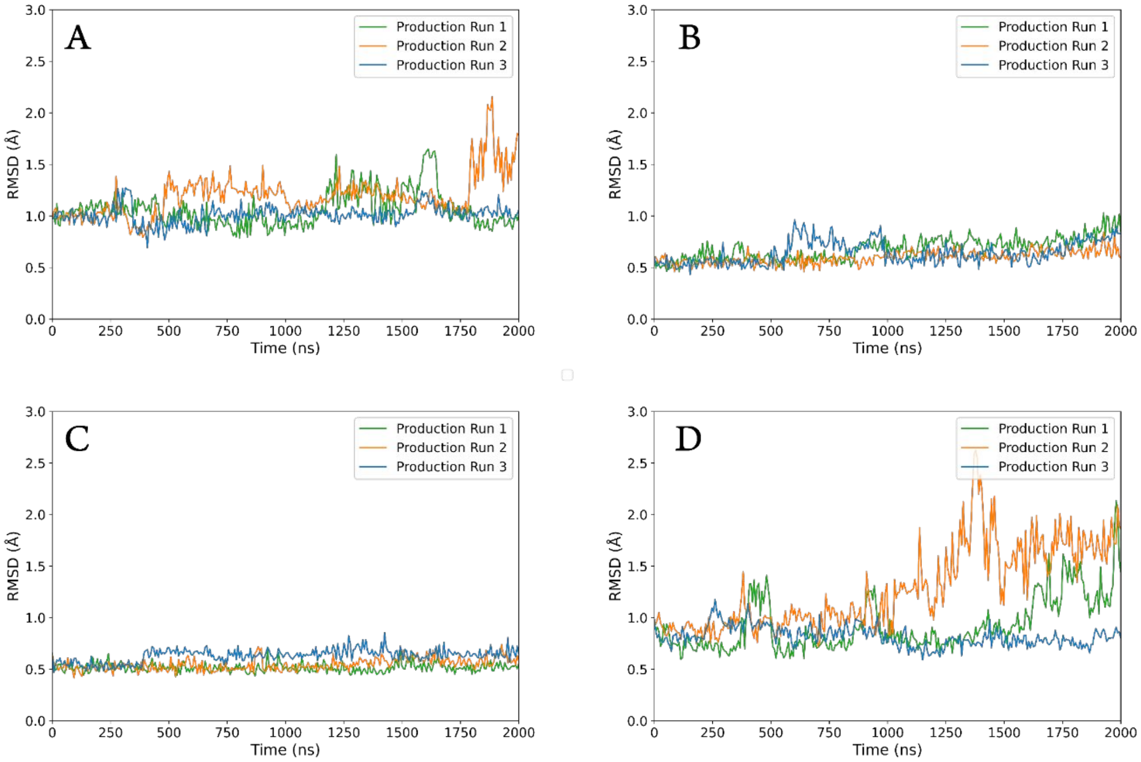

Figure S5. RMSD of PrnA active site (all residues with one atom within 7 Å of substrate Trp) relative to the average structure for protonation states A-D.

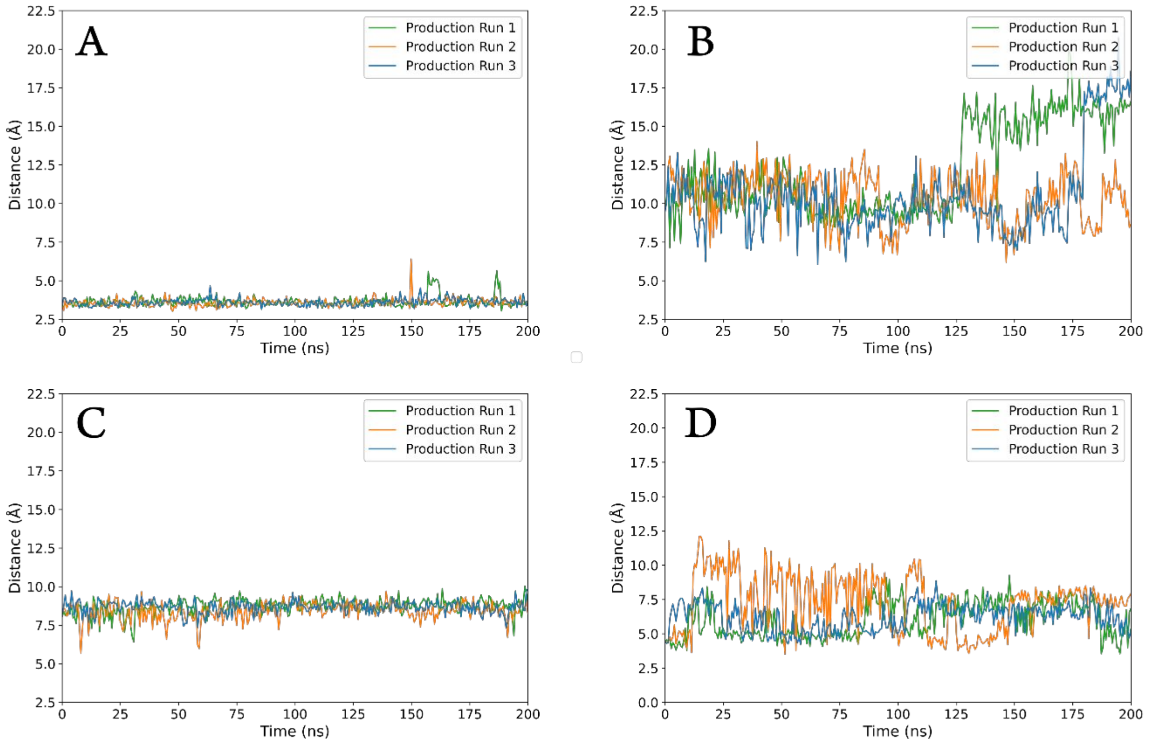

Figure S6. Trp C7-Cl distance vs time plots for protonation states A-D.

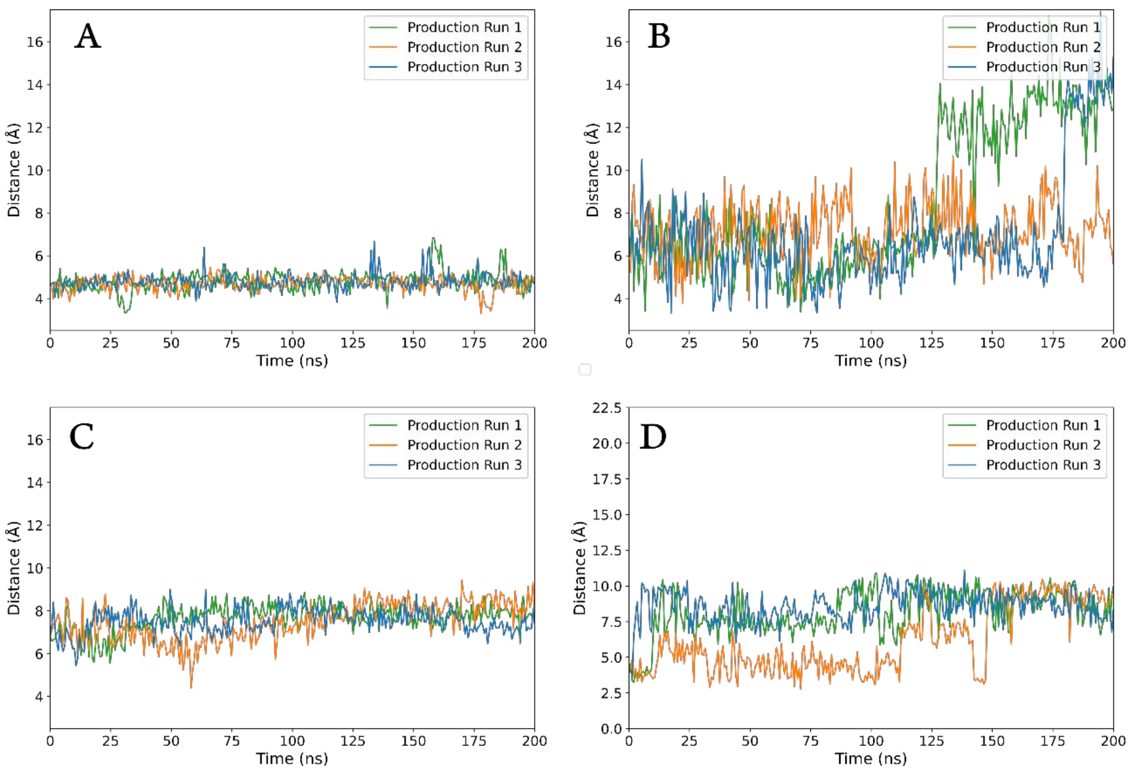

Figure S7. Lys79 N-Cl distance vs time plots for protonation states A-D

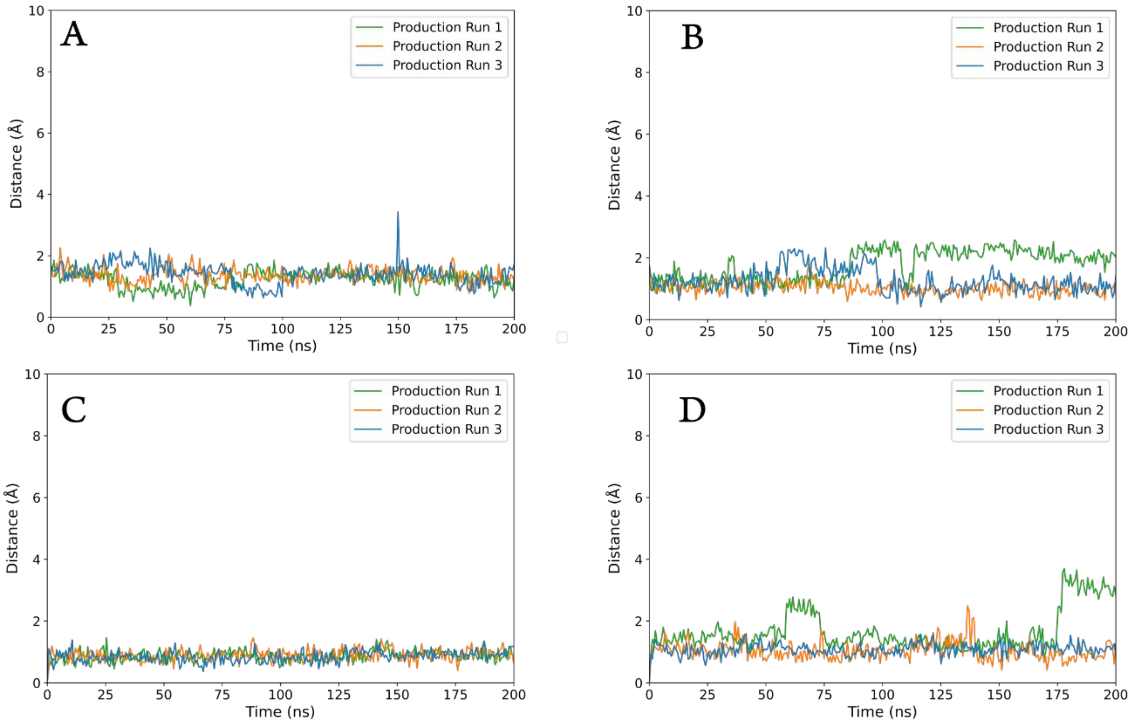

Figure S8. RMSD of substrate Trp during each MD simulation for protonation states A-D after structural alignment of the PrnA backbone .

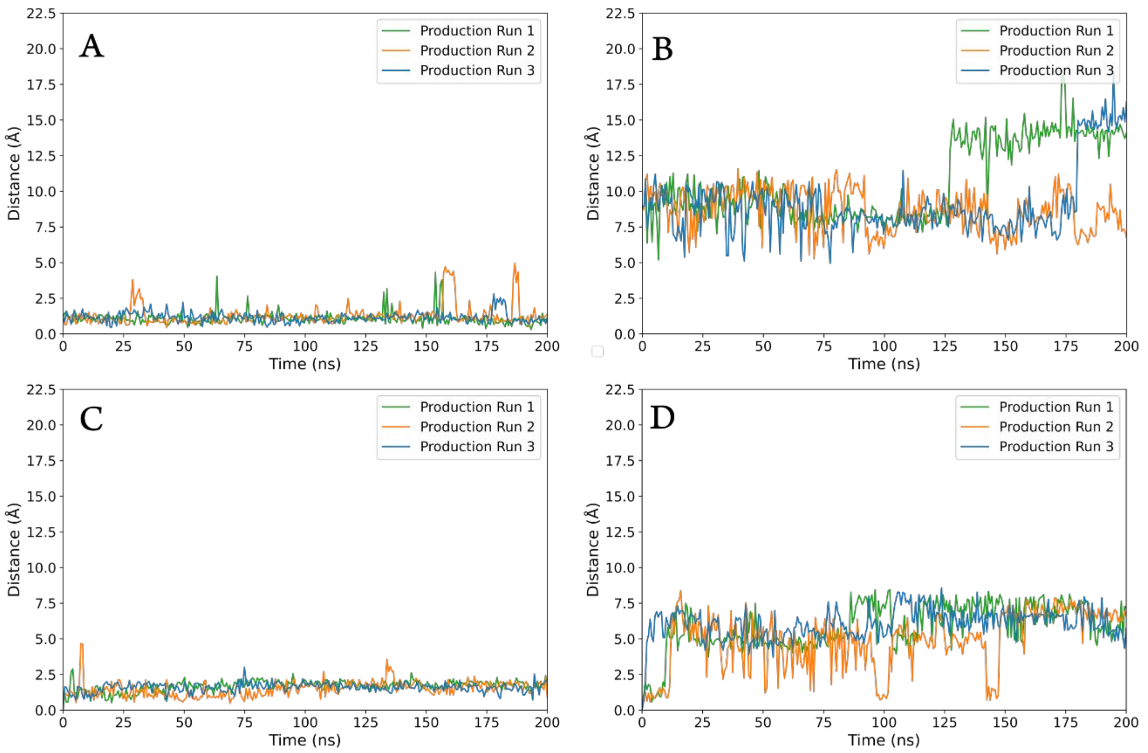

Figure S9. RMSD of HOCl during each MD simulation for protonation states A-D after structural alignment of the PrnA backbone.

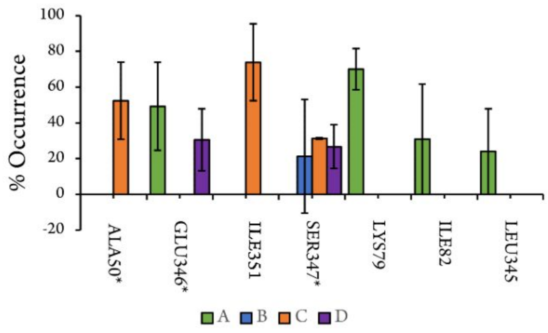

Figure S10. Average % occurrence of hydrogen bonding interactions (acceptor = *) between HOCl and PrnA for all production runs of each protonation State. For clarity only hydrogen bonds with >20% occurrence during simulations are shown. "GLH" refers to protonated Glu.

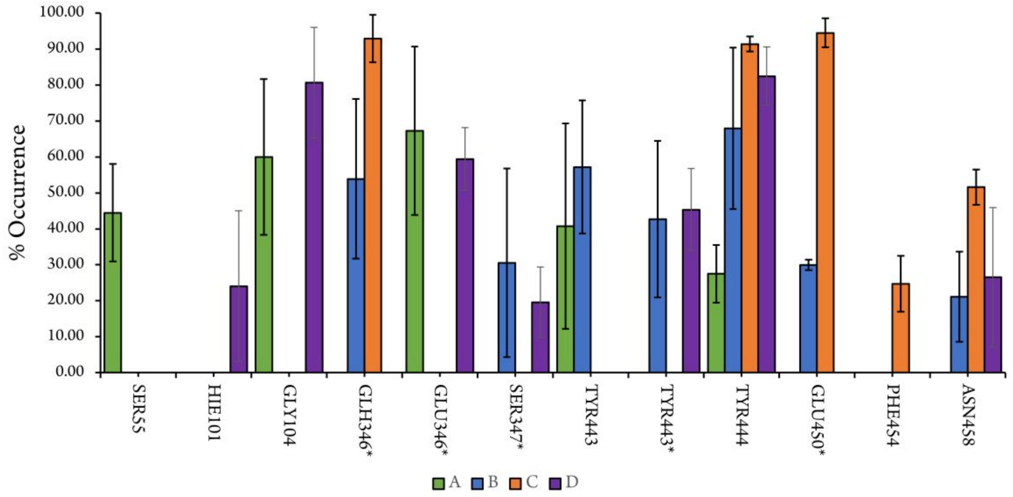

Figure S11. Average % occurrence of hydrogen bonding interactions (acceptor = *) between Trp and PrnA for all production runs of each protonation State. For clarity only hydrogen bonds with >20% occurrence during simulations are shown. "GLH" refers to protonated Glu.

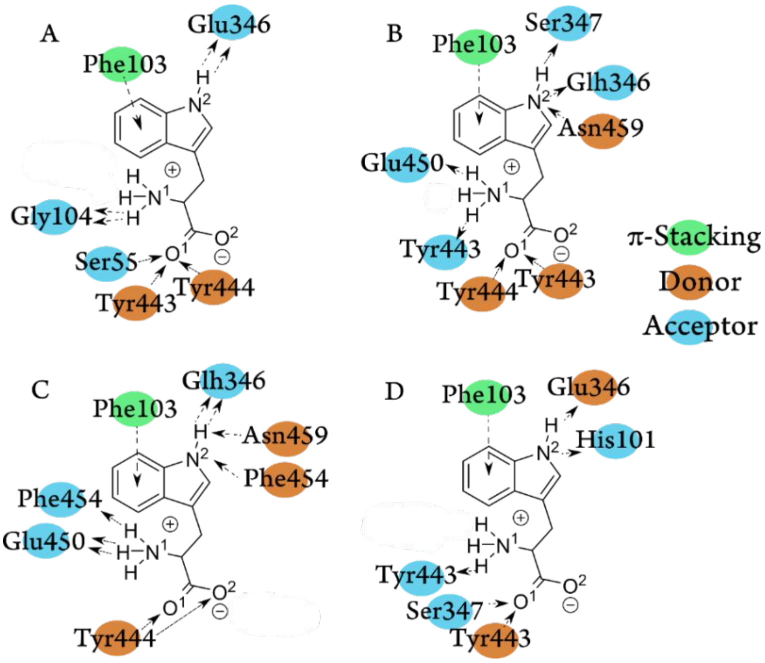

Figure S12. Illustration of the hydrogen bonding with >20% occurrence for Trp in each state. “Glh” refers to protonated Glu.

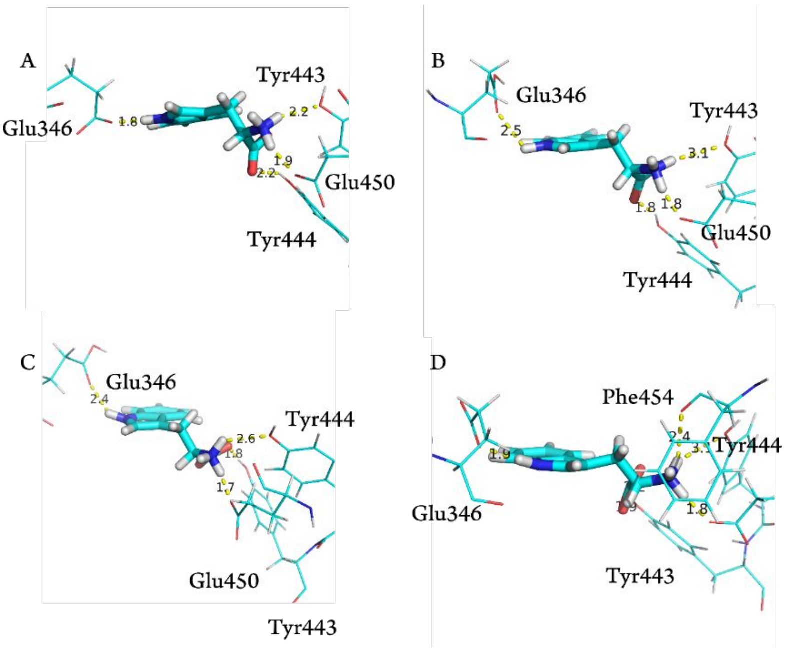

Figure S13. Hydrogen bonding to Trp in representative structure for States A-D (Panels A-D, respectively).

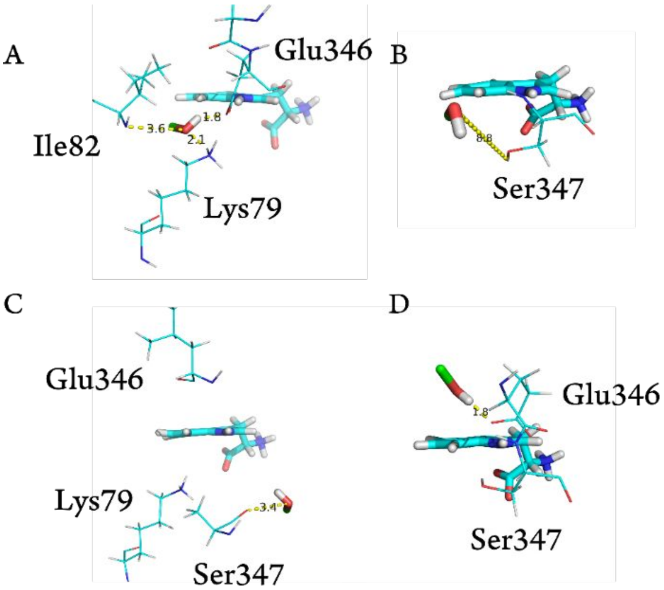

Figure S14. Hydrogen bonding to HOCl in representative structure for states A-D (Panels A-D, respectively).

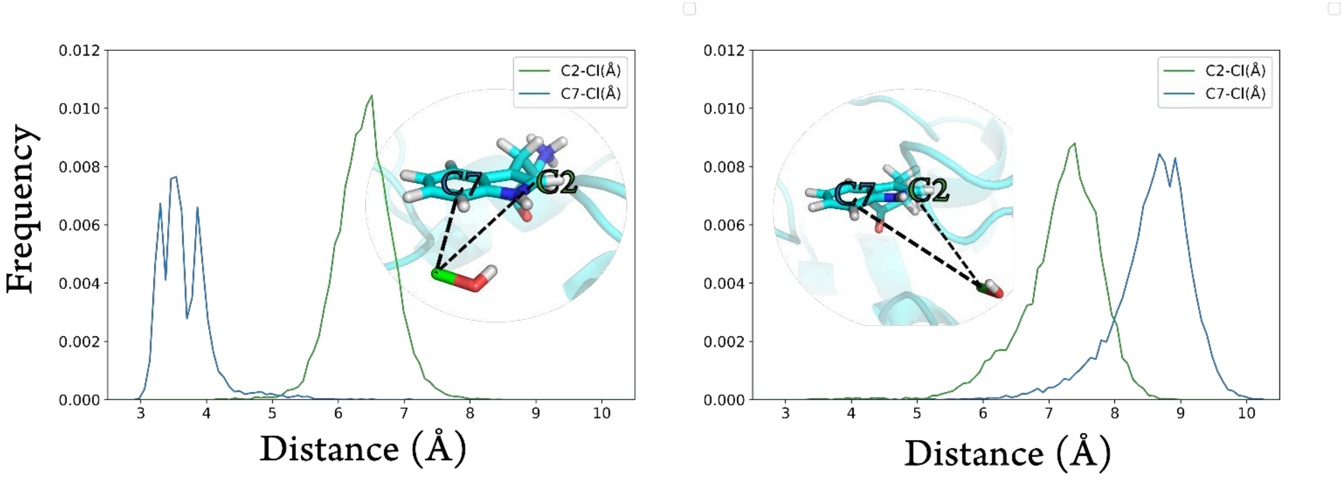

Figure S15. Distributions of C2-Cl (green) and C7-Cl (blue) distances during all MD runs of PrnA, states A (left) and C (right).

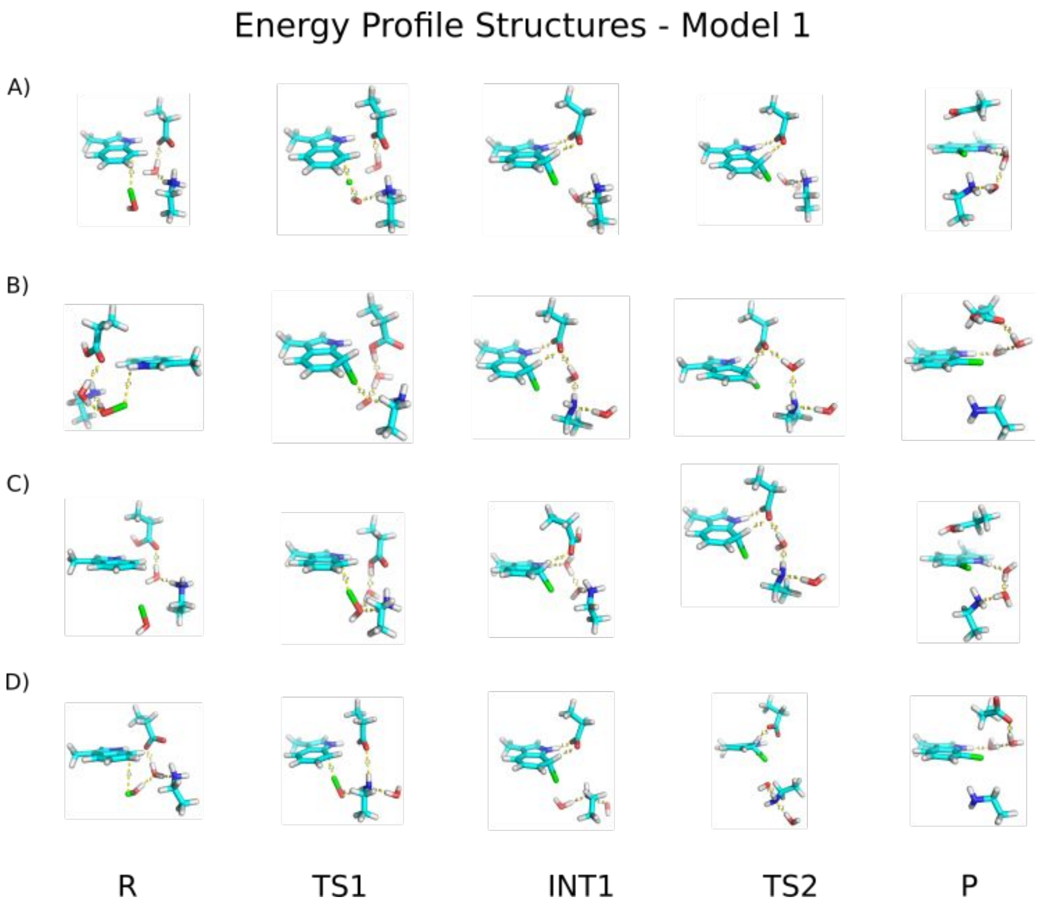

Figure S16. Structures from the reaction profile for mechanism 1 for Protonation States A-D calculated for Model 1. Labels correspond to energies in Figure 6.

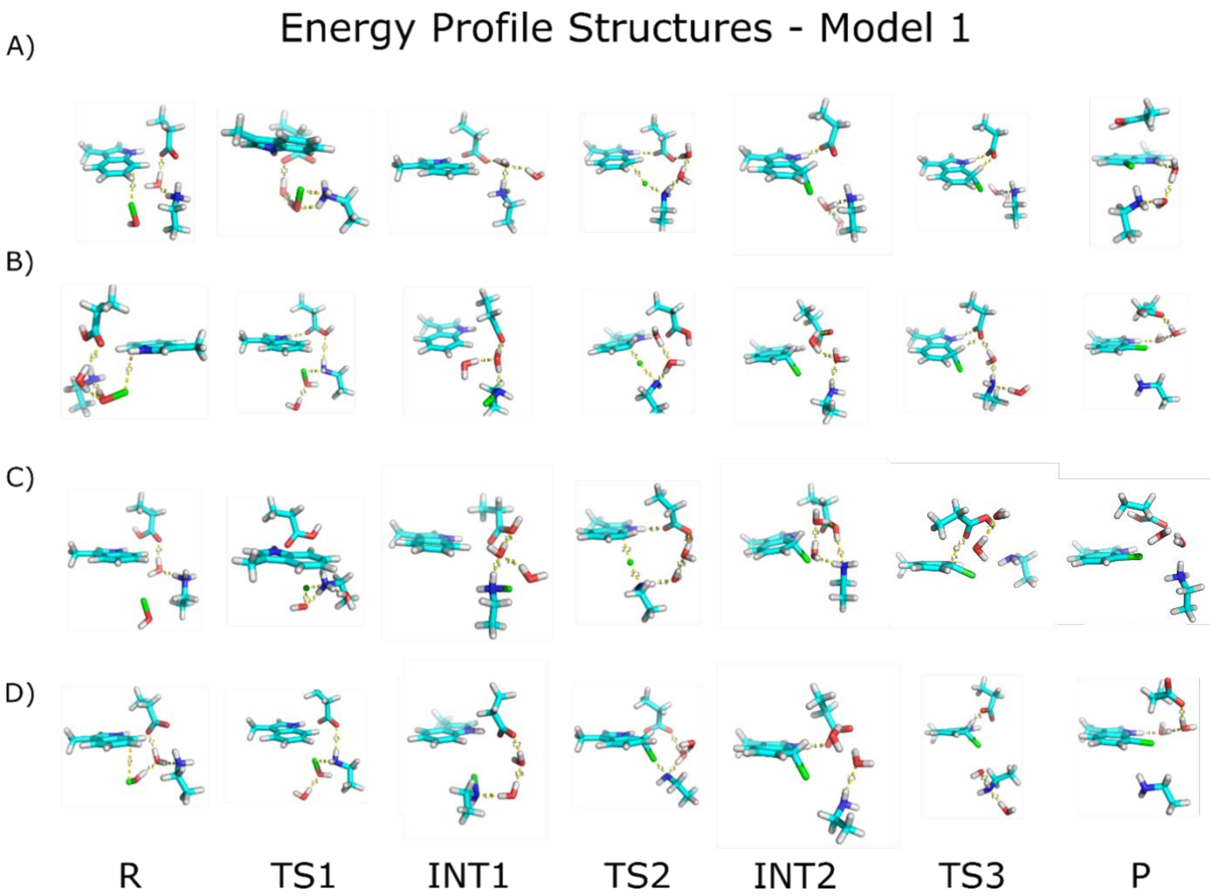

Figure S17. Structures from the reaction profile for mechanism 2 for Protonation States A-D calculated for Model 1. Labels correspond to energies in Figure 7.

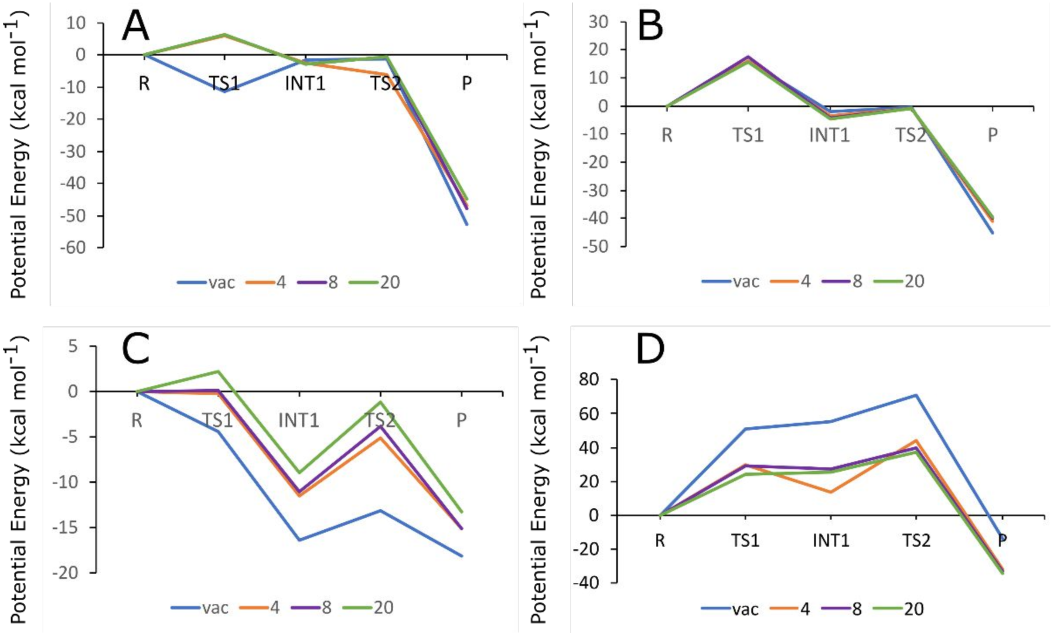

Figure S18. Energy profile of mechanism 1 in Model 1 in different dielectric constants and in vacuum.

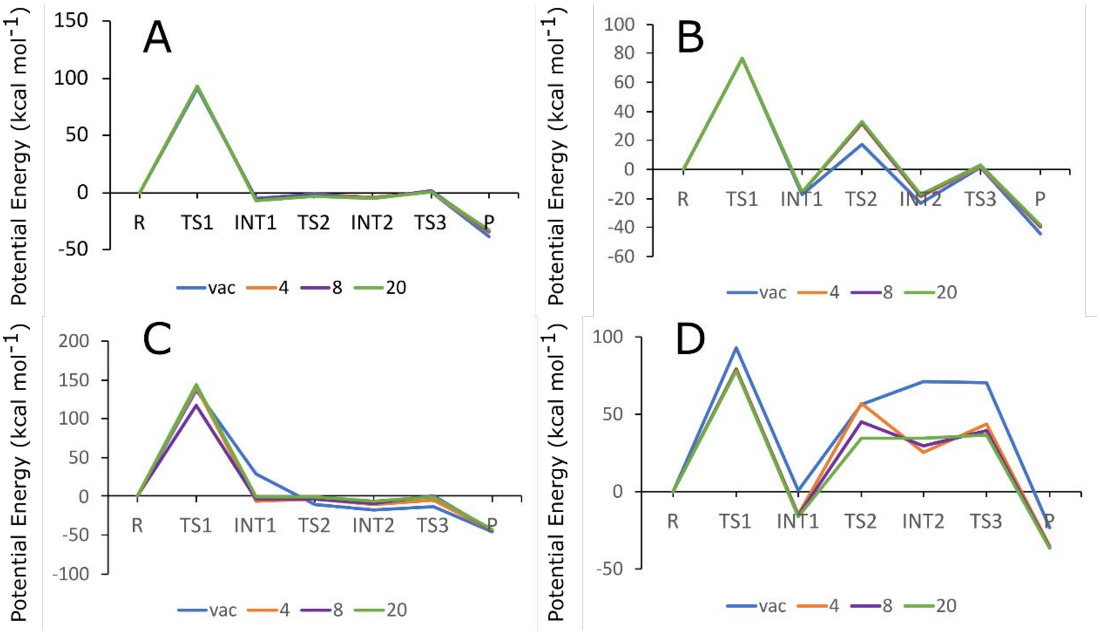

Figure S19. Energy profile of mechanism 2 in Model 1 in different dielectric constants and in vacuum.

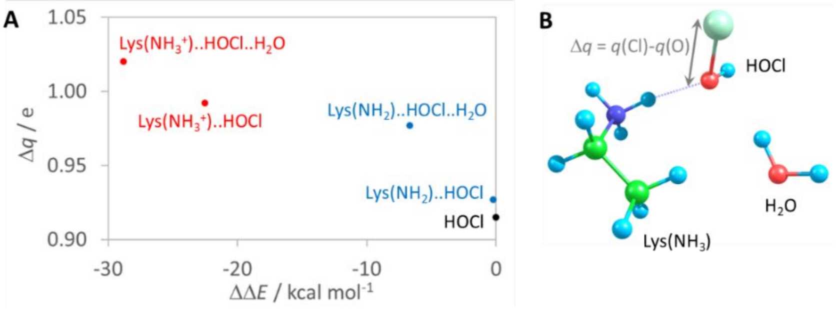

Figure S20. Effect of hydrogen bonding on O-Cl bond polarization and bond dissociation energy: (**A**) plot of the difference in the natural charges of Cl and O of HOCl (Δq, in electrons) *vs* the relative bond dissociation energy (BDE) for the O-Cl bond compared to an isolated HOCl ($\Delta\Delta E$, in kcal mol^{-1}), for isolated HOCl, HOCl hydrogen-bonded to a neutral lysine analog (Lys(NH_2)..HOCl, blue) and a lysinium analog (Lys(NH_3^+, red)..HOCl), as well as additional an water molecules (Lys(NH_2)..HOCl.. H_2O and (Lys(NH_3^+)..HOCl..H_2O); (**B**) the energy minimised Lys(NH_3^+)..HOCl..H_2O model for illustrative purposes. When adding the H_2O the remaining atoms were fixed to their energy minimized coordinates to prevent hydrogen bonding between the lysine/lysinium and H_2O, especially with neutral lysine for which the weak hydrogen bond to HOCl is otherwise broken. BDEs were calculated without re-optimising after removing the Cl^+, in order to prevent the additional bond breaking and forming associated with proton transfer from Lys(NH_3^+) to the resulting OH^-.

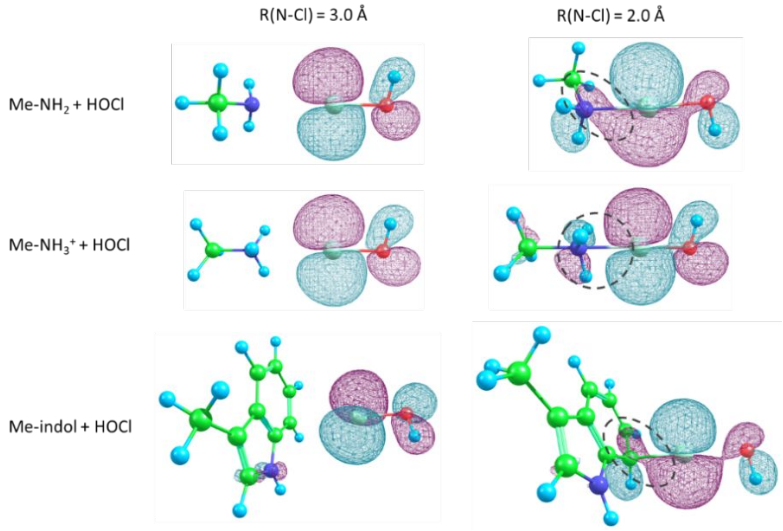

Figure S21. Reactivity of HOCl towards neutral and protonated lysine analog (Me-NH$_2$ and Me-NH$_3^+$) and tryptophan analog (Me-indol): as the Cl approaches the acceptor N and C atoms a bonding orbital forms with Me-NH$_2$ and Me-indol (as shown by dotted ovals) but not with Me-NH$_3^+$. The O-Cl-N angle was kept near linear (178°) during energy minimizations.

Model 2 Structures

Mechanism 1

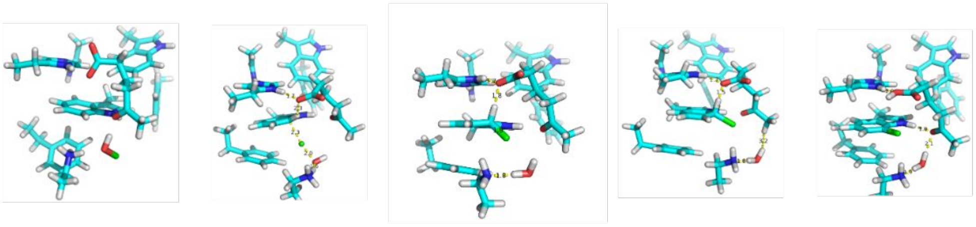

R TS1 INT1 TS2 P

Mechanism 2

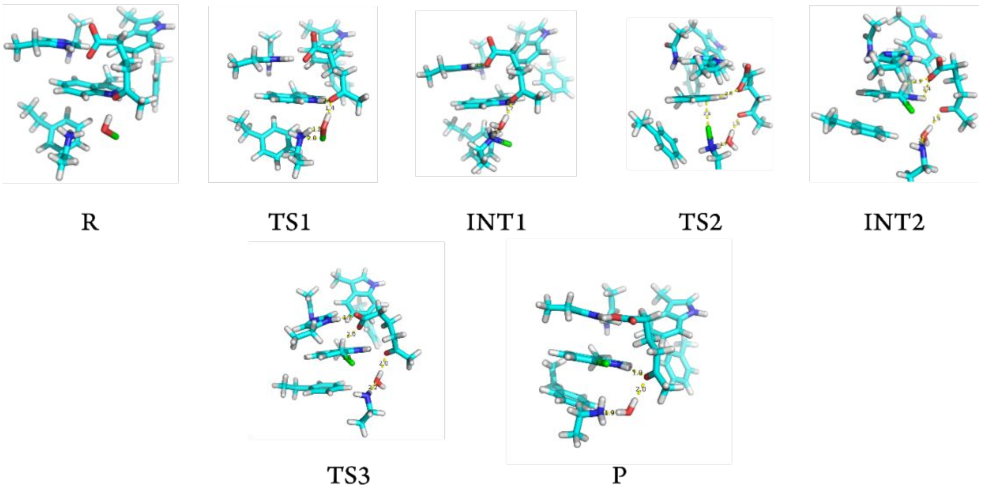

R TS1 INT1 TS2 INT2

TS3 P

Figure S22. Structures for reaction profiles of mechanism 1 for Protonation State A and 2 for Model 2. Labels correspond to energies in Figure 8.

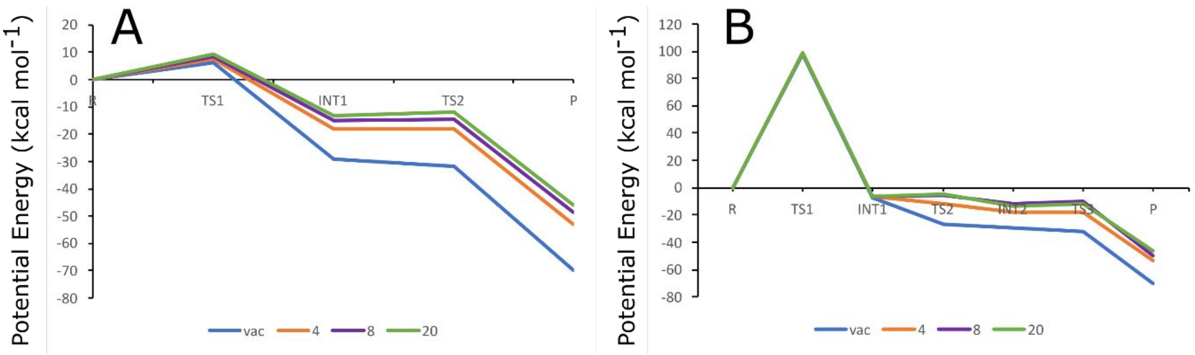

Figure S23. Energy profile of mechanism 1 in Model 2 in different dielectric constants and in vacuum.

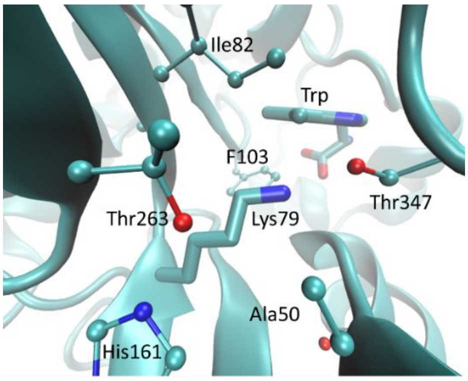

Figure S24. Residues in the first sphere around Lys79. Lys79 and substrate Trp are shown with thick bonds, while other amino acids are shown using the ball-and-stick representation.

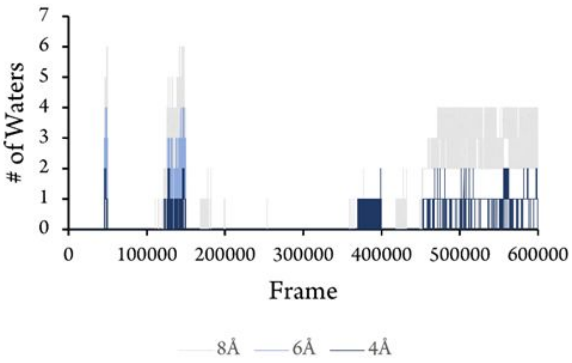

Figure S25. Number of water molecules within 4, 6 and 8 Å of Lys79 N during three MD simulations of protonation state A; each simulation consists of 200000 frames. For trajectory 1 there is only a 30 ns window where any waters come within 4A of Lys79. For trajectory 2 there is a similar window at the end of the run, and for trajectory 3 waters are inconsistently within 4A of Lys79 for 150ns, but do not form a constant proximity for longer than approximately 3ns.

Table S1. Cartesian coordinates for energy minimised reactant state of Model 3 (charge: -1, spin multiplicity: 1)

N	-6.01301	6.524566	-2.05186
H	-5.70704	5.644971	-2.45746
C	-4.87705	7.168362	-1.38799
H	-4.34672	7.88109	-2.03397
C	-5.41587	7.924079	-0.13964
H	-4.58088	8.187586	0.518296
C	-6.17678	9.189831	-0.5295
H	-5.52108	9.905211	-1.03678
H	-7.01114	8.948357	-1.19636
H	-6.58563	9.666189	0.3655
O	-6.24456	7.025345	0.584872
H	-6.73956	6.570361	-0.12476
C	-3.87169	6.09381	-0.9627
O	-4.12514	4.882693	-1.06911
N	-2.70526	6.563716	-0.49696
H	-2.5547	7.561298	-0.39453
C	-1.63145	5.749128	0.052002
H	-2.05011	4.778683	0.316932
C	-0.48543	5.552731	-0.98341
H	-0.20895	6.562271	-1.31897
C	0.755904	4.890547	-0.36735
H	0.540375	3.868075	-0.04151
H	1.564033	4.837542	-1.10139
H	1.138271	5.447486	0.491764
C	-0.98849	4.75093	-2.19935
H	-1.11251	3.70196	-1.89862
H	-1.98043	5.104598	-2.49173
C	-0.06303	4.835754	-3.41569
H	-0.44988	4.231077	-4.24226
H	0.02497	5.86946	-3.76869
H	0.946506	4.480349	-3.19169
C	-1.12823	6.508814	1.303496
O	-1.07035	7.74867	1.250524
N	-0.75177	5.81465	2.396407
C	-0.8408	4.351715	2.608634
H	-0.04978	3.825417	2.068257
H	-1.80295	3.966037	2.276891
C	-0.64097	4.20098	4.119119
H	-0.23698	3.21829	4.372162
H	-1.59668	4.322912	4.64082
C	0.308266	5.358385	4.463874
H	1.332402	5.09807	4.175711
H	0.309935	5.618306	5.524973
C	-0.20198	6.508312	3.589816
H	-1.00071	7.076334	4.081371
H	-9.71956	5.709287	1.946766
C	-8.75005	5.211321	1.847006
H	-7.96611	5.966145	1.954181
H	-8.65287	4.498556	2.67352
C	-8.63555	4.498926	0.492767
H	-9.46395	3.789808	0.370016
H	-8.74065	5.233936	-0.31696
C	-7.30153	3.760608	0.324342
H	-6.4796	4.46701	0.490223
H	-7.21135	2.976686	1.086935
C	-7.14089	3.14429	-1.06411
H	-7.8629	2.343384	-1.24216
H	-7.27539	3.902867	-1.83842
N	-5.77317	2.573705	-1.24791
H	-5.6403	1.760889	-0.63792
H	-5.56216	2.309636	-2.28436
H	-5.08243	3.300321	-1.01481
N	-9.02131	-1.52054	0.212473
H	-8.40134	-2.2562	0.54153
C	-9.29496	-1.50408	-1.21169
H	-10.0099	-0.69479	-1.41276
C	-8.06475	-1.25899	-2.15863
H	-7.61417	-0.32483	-1.79436
C	-7.02969	-2.38226	-2.02935
H	-6.82687	-2.62539	-0.98349
H	-7.38095	-3.29994	-2.51426
H	-6.07974	-2.10053	-2.48989
C	-8.49065	-1.0202	-3.61866
H	-8.90729	-1.94499	-4.0378
H	-9.3022	-0.28083	-3.63397
C	-7.34925	-0.53355	-4.51663
H	-7.71657	-0.31459	-5.52467
H	-6.88991	0.377671	-4.121
H	-6.56288	-1.28898	-4.61224
C	-9.97799	-2.79055	-1.62391
O	-9.93157	-3.82711	-0.9896
N	-7.71026	-5.73059	-0.02673
H	-8.12984	-4.80837	-0.11443
C	-6.30871	-5.61473	0.351288
H	-5.99522	-6.53902	0.850304
C	-5.31095	-5.36753	-0.82546
H	-5.76368	-5.74825	-1.74682
H	-5.18049	-4.28885	-0.97774
C	-3.98346	-6.03996	-0.63318
N	-3.40146	-6.17064	0.616935
C	-2.26158	-6.81455	0.427101
H	-1.55766	-7.08452	1.200566
N	-2.07568	-7.11032	-0.88605
H	-1.28364	-7.62029	-1.28331
C	-3.16739	-6.62457	-1.57552
H	-3.268	-6.74142	-2.64308
C	-6.1942	-4.46507	1.384772
O	-7.06278	-3.57838	1.445866

N	-5.11393	-4.49462	2.193462
H	-4.3681	-5.16779	1.977394
C	-4.97407	-3.59574	3.330002
H	-5.67425	-2.77341	3.168171
C	-5.32016	-4.31471	4.645834
H	-4.66635	-5.18153	4.777727
H	-5.17537	-3.64377	5.497308
H	-6.3589	-4.65392	4.624675
C	-3.53973	-3.05239	3.455004
O	-2.55882	-3.77219	3.270596
N	-3.45494	-1.75676	3.860199
H	-4.31572	-1.2681	4.060699
C	-2.2195	-1.13709	4.319875
H	-1.43083	-1.3778	3.603196
C	-2.3919	0.392104	4.386926
H	-3.22506	0.626923	5.060022
H	-1.48723	0.80346	4.840464
C	-2.6061	1.04225	3.038197
C	-1.65346	0.884842	2.02254
H	-0.7632	0.298455	2.20435
C	-1.80946	1.522008	0.795339
H	-1.04118	1.41252	0.041727
C	-2.92255	2.328851	0.559581
H	-3.02364	2.848536	-0.3858
C	-3.87297	2.503808	1.567751
H	-4.72308	3.160082	1.414987
C	-3.71831	1.856613	2.797095
H	-4.45748	2.001802	3.580835
C	-1.78887	-1.71013	5.683569
O	-2.53594	-2.39125	6.38624
N	-0.51906	-1.37459	6.026965
H	0.031425	-0.78854	5.3877
C	0.050727	-1.79464	7.276668
H	0.400793	-0.94239	7.878355
H	-0.73109	-2.2885	7.86693
C	1.210515	-2.75332	7.163378
O	1.687128	-3.18665	6.131835
C	-2.94966	0.999397	-6.44332
H	-2.70039	-0.00017	-6.07533
H	-3.04931	0.947572	-7.53213
C	-4.24243	1.515562	-5.81125
H	-5.08359	0.862938	-6.07953
H	-4.50236	2.501957	-6.21207
C	-4.26452	1.627747	-4.28032
O	-3.32828	1.148137	-3.59123
O	-5.30017	2.208814	-3.79793
C	9.782333	0.490822	5.909743
H	10.84392	0.511828	5.6474
H	9.626107	-0.35871	6.585679
C	8.912648	0.377366	4.674027
C	9.431448	-0.04026	3.442591
H	10.49291	-0.25867	3.35675
C	8.618398	-0.19293	2.313286
H	9.043533	-0.52608	1.370294
C	7.250488	0.076296	2.406873
O	6.380732	-0.04921	1.366248
H	6.756553	-0.39812	0.537149
C	6.711681	0.515771	3.623203
H	5.647509	0.72584	3.668311
C	7.53757	0.659579	4.733812
H	7.103802	0.995252	5.67259
N	11.08079	3.533797	1.381408
H	10.81233	3.565832	2.36192
C	9.954364	3.02665	0.595358
H	9.758149	1.951259	0.724732
C	8.671684	3.810705	0.966248
H	8.578348	3.774014	2.059307
H	8.795851	4.865669	0.704468
C	7.384249	3.289938	0.327107
H	7.547549	3.08521	-0.73797
H	7.081515	2.335269	0.767022
C	6.20994	4.294051	0.409513
O	6.461422	5.493292	0.625907
O	5.034071	3.818052	0.205527
C	10.2388	3.274522	-0.86983
O	9.884428	2.551672	-1.78334
N	9.187837	-0.36412	-2.85469
H	9.804211	0.417989	-3.0684
C	7.869789	0.183936	-2.52565
H	7.865411	0.852308	-1.65711
C	7.330917	0.945774	-3.75557
H	8.016702	1.78189	-3.93209
H	7.401072	0.276193	-4.61885
C	5.920415	1.461645	-3.60626
C	5.676152	2.710449	-3.02181
H	6.512512	3.314484	-2.68416
C	4.373786	3.190445	-2.8742
H	4.206773	4.142387	-2.38342
C	3.298069	2.425048	-3.32491
H	2.286562	2.801338	-3.21862
C	3.525206	1.17328	-3.90355
H	2.691215	0.564617	-4.2386
C	4.828685	0.6923	-4.03613
H	5.001907	-0.28577	-4.47411
C	6.908414	-0.98766	-2.14327
O	6.090964	-0.84638	-1.2178
N	6.978674	-2.12657	-2.87632
H	7.769791	-2.18193	-3.50886
C	6.195433	-3.35457	-2.62821
H	5.30455	-3.05364	-2.06369
C	5.766862	-4.02413	-3.94949

H	6.659989	-4.24021	-4.54618
H	5.316401	-4.99265	-3.69682
C	4.799402	-3.19685	-4.73392
C	5.012071	-2.59608	-5.95195
H	5.897138	-2.58901	-6.57176
N	3.862689	-1.9426	-6.35794
H	3.763561	-1.42173	-7.2147
C	2.880287	-2.10936	-5.40218
C	1.557146	-1.65287	-5.36314
H	1.143869	-1.05538	-6.17013
C	0.795045	-1.99025	-4.24915
H	-0.22774	-1.63732	-4.17441
C	1.336311	-2.75655	-3.19502
H	0.723077	-2.97237	-2.32825
C	2.651346	-3.20293	-3.23189
H	3.056599	-3.75415	-2.38897
C	3.442104	-2.89139	-4.35312
C	6.959259	-4.3199	-1.73804
O	8.066166	-4.11475	-1.28976
N	1.553304	-7.80992	1.199735
H	2.448334	-8.28964	1.272226
C	1.391719	-7.17551	-0.11687
H	0.435718	-6.64539	-0.11942
C	2.513391	-6.15969	-0.38439
H	3.494837	-6.63986	-0.27855
H	2.44972	-5.80848	-1.41786
C	2.501684	-4.90309	0.493394
O	3.076715	-3.8777	0.081839
N	1.865913	-4.99438	1.667846
H	1.758008	-4.18001	2.282039
H	1.532748	-5.90964	1.961298
C	1.335444	-8.23731	-1.19547
O	0.320294	-8.53664	-1.80336
O	3.302038	0.46348	2.192738
C	2.50036	1.139838	1.484297
O	1.426561	1.667693	1.864982
C	2.882539	1.258062	-0.01167
N	4.369453	1.352127	-0.0769
C	2.412267	0.007799	-0.78673
C	0.949034	-0.06971	-1.11834
C	0.265976	0.639558	-2.08788
N	-1.00884	0.13165	-2.24641
C	-1.17905	-0.92112	-1.37535
C	0.025193	-1.05847	-0.62624
C	0.107724	-2.057	0.361503
C	-0.98232	-2.89376	0.568459
C	-2.15161	-2.77686	-0.21735
C	-2.26186	-1.7965	-1.20738
H	2.460523	2.162415	-0.45062
H	2.984433	-0.03851	-1.71863
H	2.699825	-0.87917	-0.21341
H	0.599772	1.457318	-2.70792
H	1.001495	-2.16668	0.963632
H	-0.9517	-3.64124	1.351509
H	-2.96612	-3.47384	-0.05426
H	-3.13595	-1.72977	-1.84592
H	-1.74796	0.526202	-2.83039
H	4.724156	2.401068	0.031025
H	4.7608	0.948077	-0.93105
H	4.753339	0.805829	0.708528
Cl	-4.6198	-0.79622	0.259602
O	-6.12353	0.04502	0.575407
H	-6.81003	-0.61426	0.348743
O	3.904515	-2.17472	2.164282
H	3.668906	-2.6379	1.333922
H	3.797516	-1.20756	2.012031
O	0.865094	-0.01752	4.015279
H	1.079251	0.688446	3.361863
H	1.159269	-0.85151	3.595712
O	1.588585	-2.62844	3.335845
H	2.528668	-2.4425	3.052374
H	1.603835	-2.85477	4.284054
H	1.636522	-3.05392	8.141631
H	2.276957	-8.79463	-1.37311
H	0.820767	-8.49343	1.372729
H	-7.79152	-6.19024	-0.92861
H	10.78791	4.221217	-1.06447
H	11.86208	2.884722	1.325423
H	9.584487	-0.79936	-2.02364
H	6.408117	-5.25827	-1.51647
H	-10.5175	-2.75172	-2.59118
H	-8.80794	-0.6118	0.601953
H	-6.37056	7.108357	-2.80018
H	9.554083	1.39955	6.476058
H	0.573799	7.214424	3.285728
H	-2.10533	1.656134	-6.211
